# Comprehensive immunophenotypic analysis reveals the pathological involvement of Th17 cells in Graves' disease

**DOI:** 10.1038/s41598-022-19556-z

**Published:** 2022-10-07

**Authors:** Keiichi Torimoto, Yosuke Okada, Shingo Nakayamada, Satoshi Kubo, Akira Kurozumi, Manabu Narisawa, Yoshiya Tanaka

**Affiliations:** First Department of Internal Medicine, School of Medicine, University of Occupational and Environmental Health, 1-1 Iseigaoka, Yahatanishi-ku, Kitakyushu-shi, 807-8555 Japan

**Keywords:** Autoimmunity, Thyroid diseases, T-helper 17 cells

## Abstract

Graves' disease (GD) is an organ-specific autoimmune disease, but there are a few studies that have evaluated how immunophenotypes are related to clinical symptoms and intractable pathology, or the effects of treatment on immunophenotypes. We performed peripheral blood immunophenotyping in GD. We assessed the proportion of functional subsets of T helper cells (such as Th1, Th17, Treg and Tfh cells), B cells (Naïve, IgM memory, Class-switched, IgD^−^CD27^−^ double negative and Plasmablasts cells), Monocytes, Dendritic cells and NK cells, and evaluated the relationship of immunophenotypes with clinical indices, disease activity, risk of relapse, and changes in immunophenotypes after treatment with antithyroid drugs. The activated Th17 cells, activated T follicular helper (Tfh) cells, and IgD^−^CD27^−^ double-negative B cells were higher in newly onset GD compared with healthy participants. Th17 cells were associated with thyroid autoantibodies, thyroid function, thyroid enlargement, and Graves' Recurrent Events After Therapy (GREAT) score; while double-negative B cells were associated with thyroid autoantibodies. Treatment with antithyroid drugs decreased the activated Tfh cells in parallel with the improvement in thyroid function. However, activated Th17 cells were not associated with clinical improvement and remained unchanged. Peripheral blood immunophenotyping identified the differential involvement of T and B cell subsets in the pathogenesis of GD. Abnormalities in the differentiation of Th17, Tfh, and double-negative B cells reflected the clinical pathology associated with autoantibody production and excess thyroid hormones. And Th17 cells are significantly associated with the marker for resistance to treatment. These results suggest the involvement of Th17 cell activation in the intractable pathology associated with potential immune abnormalities in GD.

*Clinical trial registration*: #UMIN000017726 (Date: June 1st, 2015).

## Introduction

Graves' disease (GD) is the most common cause of hyperthyroidism in iodine sufficient areas. Antibodies against thyroid stimulating hormone (TSH) receptor in the blood persistently stimulate TSH receptors expressed on thyroid follicular cells, leading to diffuse enlargement of the thyroid gland with characteristic clinical signs and symptoms. Although methimazole and propylthiouracil are available as antithyroid drugs, treatment with methimazole is the first-line drug^1^, except in early pregnancy. Treatment with antithyroid drugs (ATD) is effective in about 60% of the cases^2,3^. Thyroid gland enlargement and high levels of thyrotropin receptor autoantibodies (TRAb) are common, especially in intractable cases^4^.

GD is an organ-specific autoimmune disease. As for other autoimmune disorders, GD likely results from the breakdown in the immune tolerance mechanisms, both at systemic and local levels^5^. Failure of T regulatory cell activity^6^, proliferation of autoreactive T and B cells^7,8^ and decrease of NK cells drive the development of the disease^5^. T helper (Th) 1 and Th2 cell subsets and an emerging role of Th17^9^ and Th22^10^ cell have been implicated in GD pathogenesis. Among these immune cells, Th17 cells, which are associated with intractable pathologies^11^, and Tfh cells, which are associated with autoantibody production, are of interest. The thyroid gland is infiltrated with CD4^+^ and CD8^+^ lymphocytes^12^, together with high expression of immunocompetent cells, such as Th17^11^ and Tfh^13^ cells. However, treatment that specifically interferes with the immune mechanism of GD is generally not yet available. Furthermore, symptomatic treatment with ATDs often leads to disease relapse, and thus, direct treatment of the pathology is desired. In this regard, immunophenotyping to identify the immune cells involved in the pathogenesis and treatment, is imperative.

Previous studies examined peripheral blood (PB) immunophenotypes in patients with GD, but none evaluated how immunophenotypes are related to the clinical symptoms and intractable pathology, or how these cells change after treatment. We reported previously that PB immunophenotyping is useful for predicting the pathogenesis, therapeutic response, and therapeutic efficacy in patients with rheumatoid arthritis^14^. In the present study, we evaluated PB immunophenotypes in patients with newly onset GD before and after treatment, and examined the relationship between immunophenotypes and clinical findings, refractory pathology and treatment response. Based on this approach, the aim of this study was to determine the changes in the phenotype of peripheral immune cells in the GD patients and their potentially attempting to predict the response to treatment.

## Results

### Baseline characteristics

Table 1 summarizes the baseline clinical characteristics of the study participants. The median age of patients with newly onset GD was 44.0 years. The median levels of free triiodothyronine (fT3), free thyroxine (fT4), TRAb, and thyroid-stimulating antibody (TSAb) were 11.5 pg/mL, 4.0 ng/dL, 8.0 IU/L, and 529.0%, respectively.

**Table 1:** Baseline clinical characteristics of study participants.

	Healthy control (n=46)	Graves' disease (n=117)	*p* value
Age, years	35.0 [49.0, 65.0]	44.0 [32.0, 44.0]	0.189
Sex, n (% female)	34 (73.9%)	86 (73.5%)	0.594
Ophthalmopathy, n (%)		20 (17.1%)	
Smoking, n (%)		36 (30.7%)	
Free triiodothyronine (fT3), pg/mL		11.5 [6.8, 21.8]	
Free thyroxine (fT4), ng/dL		4.0 [2.6, 6.8]	
TRAb, IU/L		8.0 [3.9, 19.6]	
TSAb, %		529.0 [225.5, 1323.0]	
TgAb, IU/mL		60.0 [19.8, 436.8]	
TPOAb, IU/mL		61.0 [14.0, 283.0]	
Right lobe size on ultrasoud, mm		16.0 [14.0, 19.0]	
Left lobe size on ultrasoud, mm		16.0 [13.3, 18.0]	
Isthmus size on ultrasound, mm		3.0 [2.0, 4.0]	
Velocity in right STA, cm/s		65.0 [45.9, 88.0]	
Velocity in left STA, cm/s		61.0 [44.0, 87.0]	
GREAT score, points		3.0 [1.0, 4.0]	
GREAT class, classI/classII/classIII		31 (26.5%)/47 (40.2%)/39 (33.3%)	

Data are median [Q1, Q3] or n (%). *p* values by Fisher’s exact test, t-test or Mann–Whitney U-test. The reference ranges at our hospital are as follows: TRAb ≤ 2.0 U/ml, TSAb ≤ 120%, TgAb < 28.0 IU/ml, and TPOAb < 16 IU/ml.

Ultrasonography was performed using APLIO XG SSA-790A (Toshiba Medical Systems Corporation). Thyroid blood flow was measured using peak systolic velocity (PSV) of the superior thyroid artery (STA). The size of the thyroid gland was scanned transversely so that the anterior and posterior diameters of both lobes and isthmus were at their maximum.

TRAb, thyroid stimulating hormone receptor antibody; TSAb, thyroid stimulating hormone receptor-stimulating antibody; TgAb, thyroglobulin autoantibodies; TPOAb, thyroid peroxidase antibody; STA, superior thyroid artery; GREAT, Graves’ Recurrent Events After Therapy.

### High proportion of Th17 cells, Tfh cells, double-negative B cells, and plasmacytoid DC

**Figure 1:**
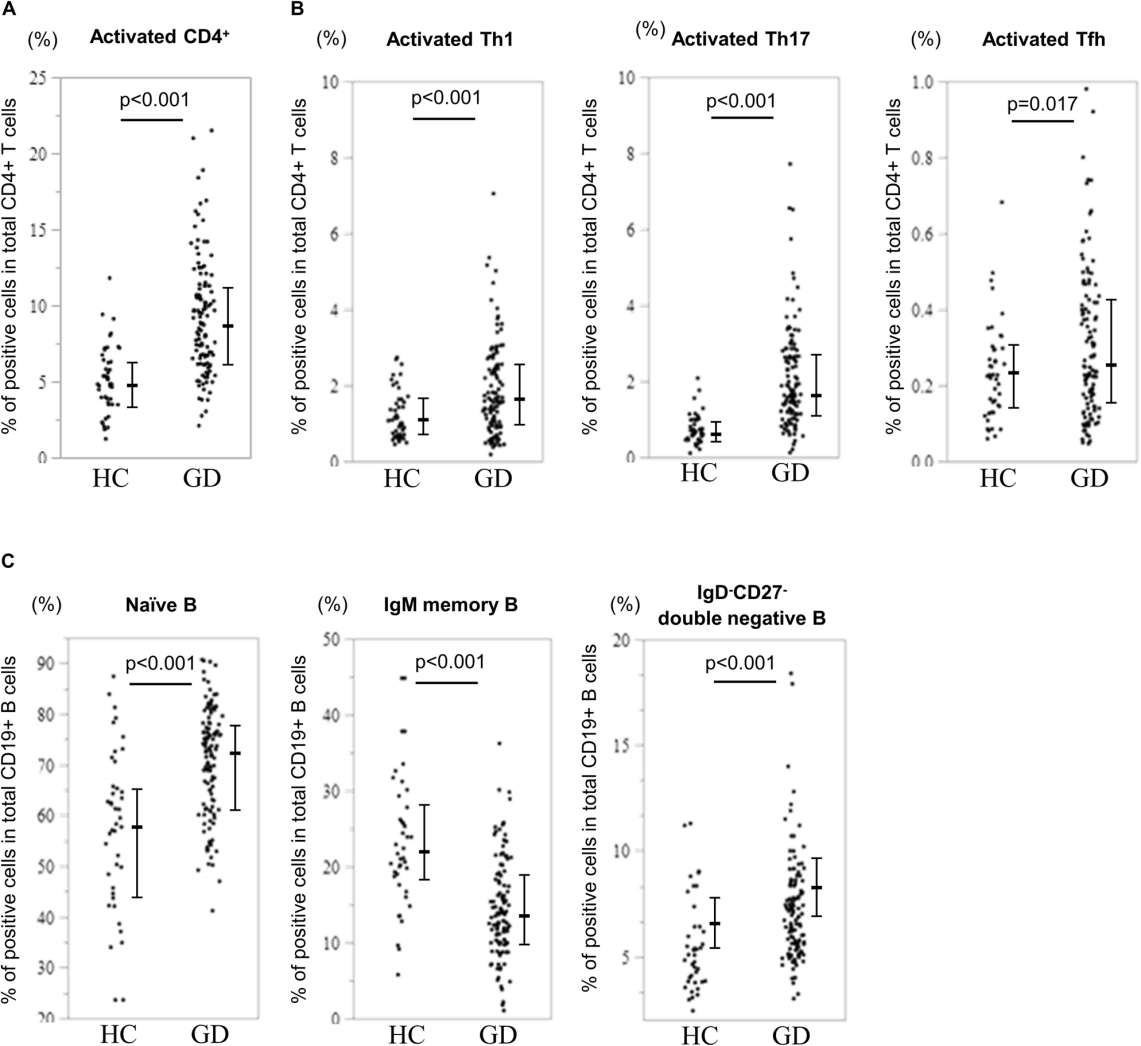
Differences in the proportions of (**A**) T helper cells, (**B**) subsets of T cells, and (**C**) B cells at baseline between patients with Graves’ disease (GD, *n* = 117) and healthy control (HC, *n* = 46) subjects. The statistical difference is determined using Student’s *t*-test for data with normal distribution, and the Mann–Whitney U test for data with skewed distribution. Th1, T helper 1 cells; Th17, T helper 17 cells; Tfh, follicular helper T cells.

Figure 1 and Supplementary Table S1 online show the results of PB immunophenotyping in both patients with newly onset GD and healthy controls (HCs). The proportion of activated Th1, activated Th17, and activated Tfh cells were elevated in patients with newly onset GD. Moreover, GD patients had lower levels of IgM memory B cells and higher levels of IgD^-^CD27^-^ double-negative B cells than HCs. In addition, the percentage of plasmacytoid dendritic cells (DCs) was significantly higher in GD patients than in HCs. We also assessed the association between these PB immunophenotypes and found that frequency of activated Th17 cells was correlated with frequency of activated Th1 cells (Supplementary Fig. S1 online).

### Correlation between Th17 cells and double-negative B cells and clinical features

**Figure 2:**
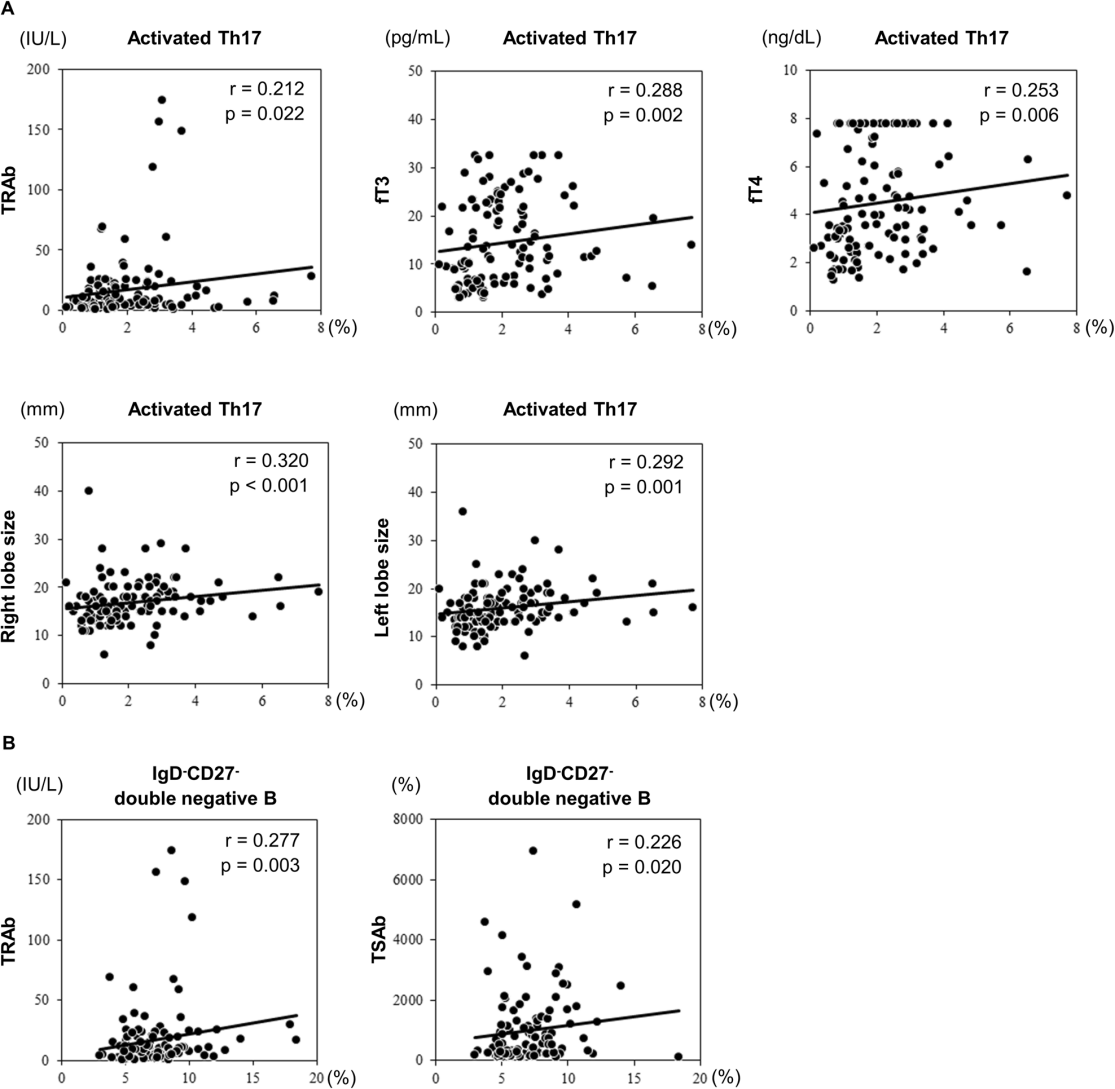
Correlations between clinical features and peripheral cell phenotype at baseline. Data are analyzed using Spearman’s correlation analysis. TRAb, thyroid stimulating hormone receptor antibody; TSAb, thyroid stimulating hormone receptor-stimulating antibody; fT3, free triiodothyronine; fT4, free thyroxine; Th17, T helper 17 cells.

Figure 2 and Supplementary Table S2 online shows the relationship of immunophenotypes with clinical characteristics. The percentage of activated Th17 cells, which was higher in the patients compared to HCs, correlated with TRAb, fT3 and fT4, and anterior–posterior diameter of both thyroid lobes. Furthermore, the percentage of double-negative B cells correlated with TRAb and TSAb.

**Figure 3:**
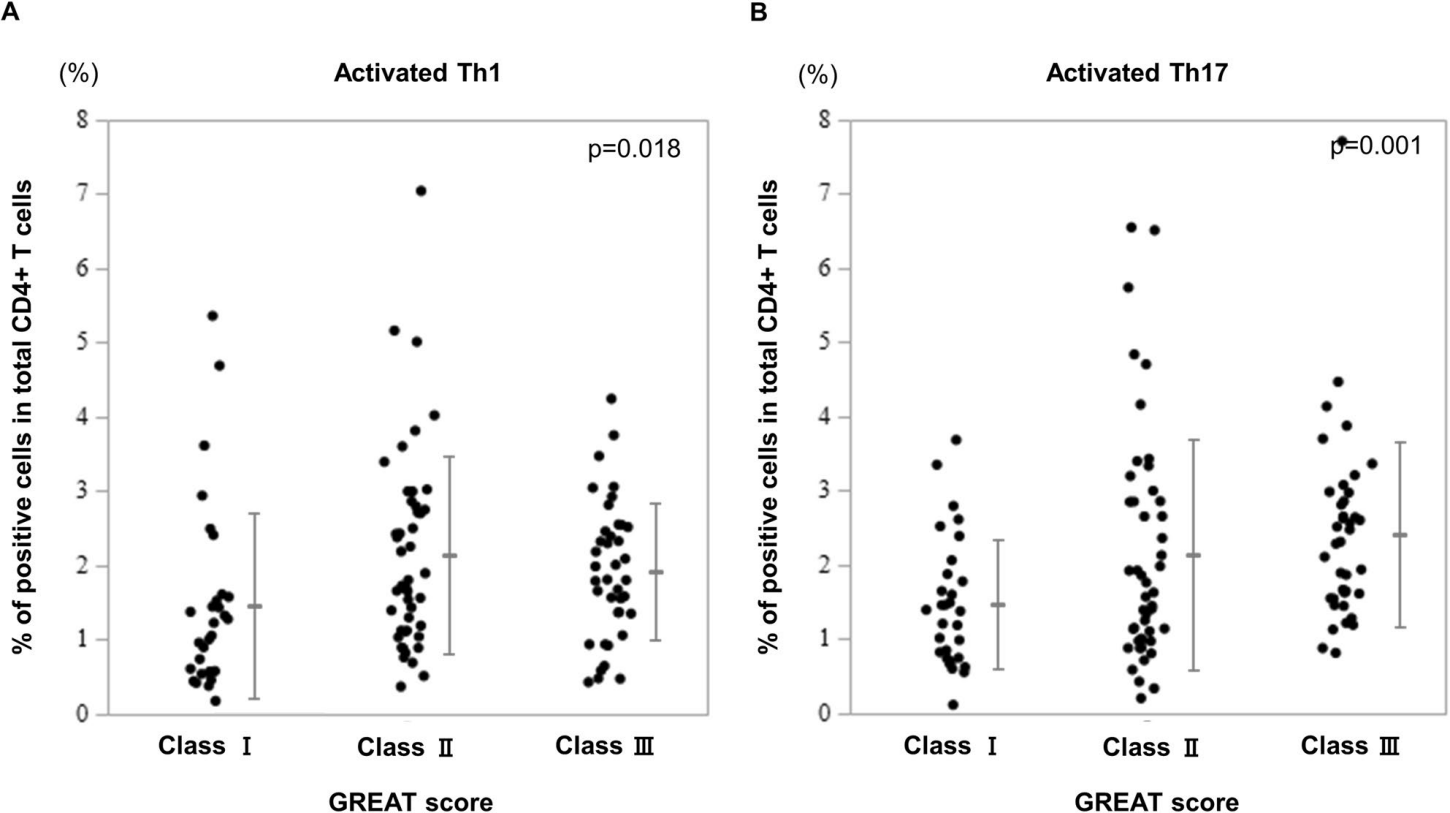
The proportions of (**A**) activated Th1 cells and (**B**) activated Th17 cells in patients per GREAT score. The statistical significance is determined by the Jonckheere–Terpstra test. GREAT, Graves’ Recurrent Events After Therapy; Th1, T helper 1 cells; Th17, T helper 17 cells.

The graves' recurrent events after therapy (GREAT) score correlated with the proportion of activated Th17 and Th1 cells. Furthermore, the proportion of activated Th17 cells increased with worsening of the GREAT classification (Fig. 3).

### Antithyroid drugs differentially change peripheral immune cell phenotypes

We explained to all patients about antithyroid drugs, total thyroidectomy and radioactive iodine, a total of 93 patients opted for treatment with ATDs, and immunophenotypic data before and after 24-week treatment were available in 79 patients (methimazole; *n* = 75, propylthiouracil; *n* = 4). 75 patients under methimazole were analyzed. Clinical parameters were evaluated before and at 24 weeks after the start of treatment. After 24-week treatment, 51 (69%) patients achieved euthyroid status. Meanwhile, 22 (30%) patients showed normal TRAb (≤ 2.0 U/ml), a significant reduction in blood flow in the superior thyroid artery; but no change in thyroid gland size (Supplementary Table S3 online).

**Figure 4:**
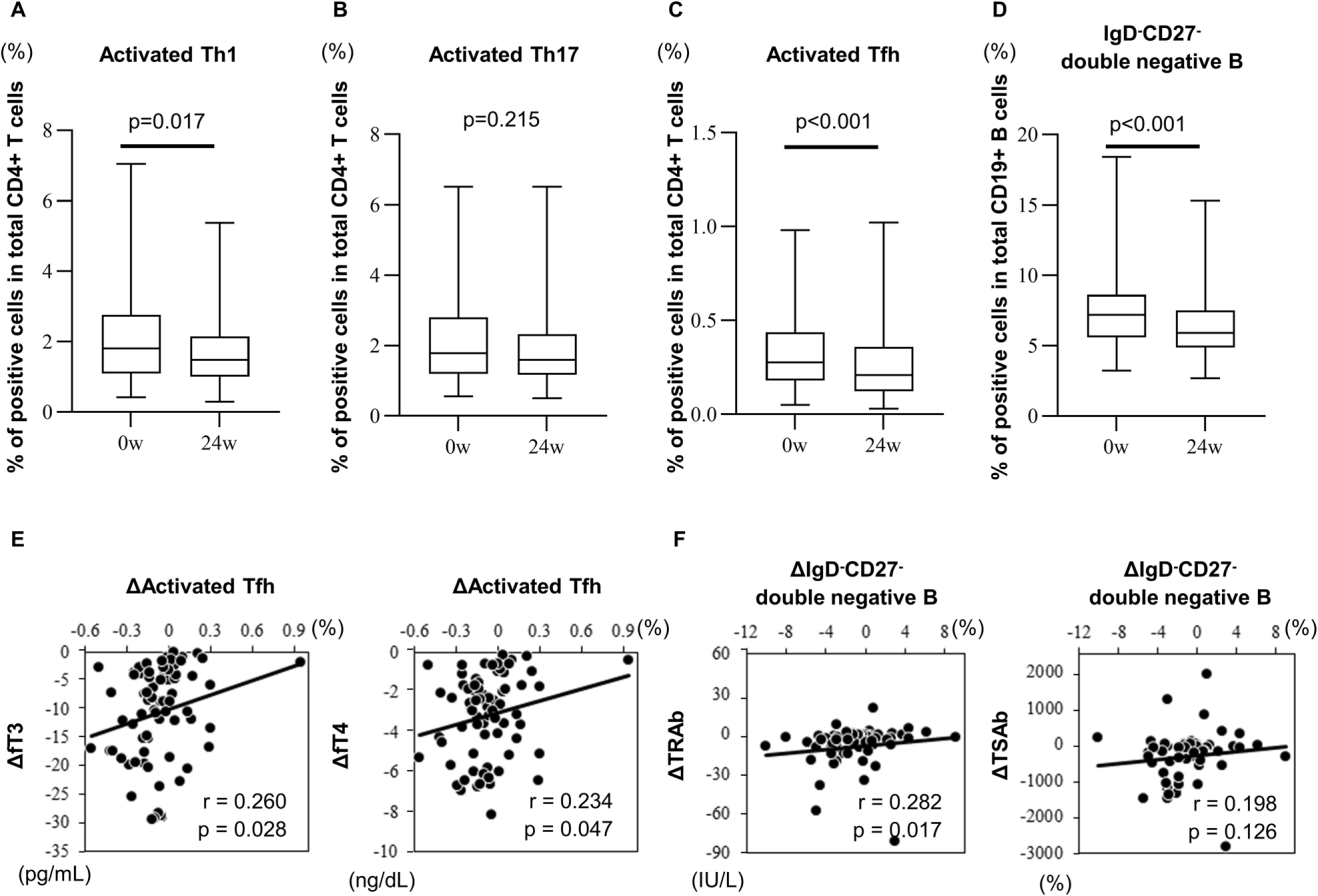
(**A**–**D**) Effects of treatment with antithyroid drugs on the proportions of peripheral immune cell phenotypes in patients with Graves’ disease. The statistical significance is determined by the paired *t*-test for data with normal distribution, and the Wilcoxon signed-rank test for data with skewed distribution. (**E**, **F**) Correlations between changes in clinical features and changes in peripheral cell phenotype. Th1, T helper 1 cells; Th17, T helper 17 cells; Tfh; follicular helper T cells; TRAb, thyroid stimulating hormone receptor antibody; TSAb, thyroid stimulating hormone receptor-stimulating antibody; fT3, free triiodothyronine; fT4, free thyroxine; Tfh; follicular helper T cells.

Furthermore, the proportions of activated Th1 cells, activated Tfh cells, and double-negative B cells, which were higher than normal before treatment, decreased after normalization of the thyroid function (*p* = 0.017, *p* < 0.001, *p* < 0.001, each) (Fig. 4 and Supplementary Table S4 online). Moreover, the decrease in thyroid function correlated with reduction in the proportion of activated Tfh cells, and the decrease in TRAb titers correlated with reduction in levels of double-negative B cells (Fig. 4 and Supplemental Table S6 online). However, the percentage of activated Th17 cells did not correlate with changes in thyroid function (Supplementary Table S6 online), and remained unchanged after treatment (Fig. 4 and Supplementary Table S6 online). Supplementary Tables S5 and S7 online show the actual numbers of PB lymphocytes. After treatment, the percentage of activated Th17 cells correlated only with anterior–posterior diameter of both the thyroid lobes, but not with TRAb, fT3, or fT4 (Supplementary Table S8 online).

### Prediction of response to antithyroid drugs by baseline peripheral immunophenotyping

**Figure 5:**
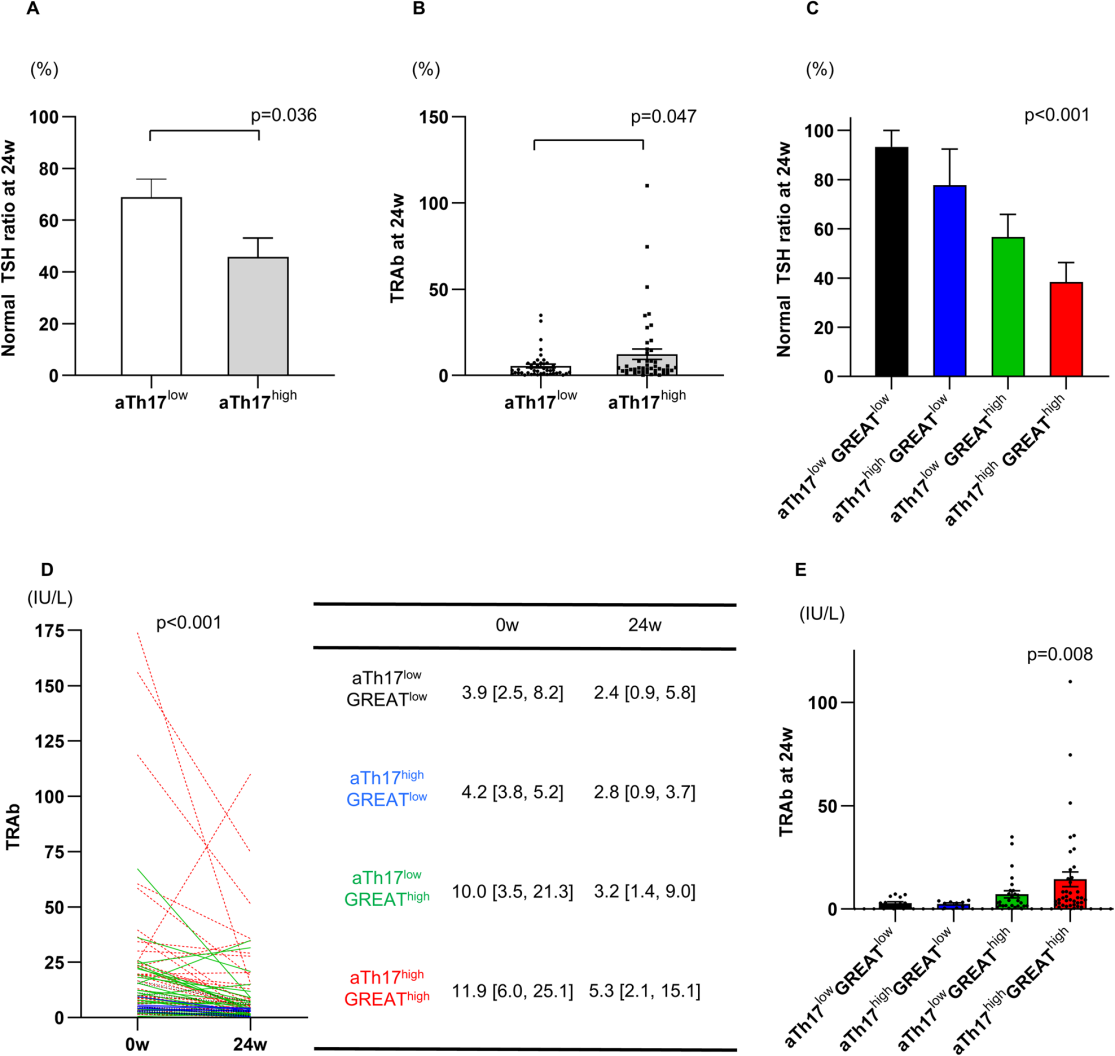
(**A**, **B**) Effects of 24-week treatment with antithyroid drugs on clinical parameters by peripheral immunophenotyping analysis and GREAT score at baseline. (**C**–**E**) Prediction of response to antithyroid drugs by peripheral immunophenotyping analysis and GREAT score at baseline. Patients are classified into four groups: activated Th17^low^/GREAT score^low^ (*n* = 15), activated Th17^high^/GREAT score^low^ (*n* = 9), activated Th17^low^/GREAT score^high^ (*n* = 30), and activated Th17^high^/GREAT score^high^ (*n* = 39). The activated Th17 cells cutoff is 1.67%; and GREAT score cutoff is 2 points. TRAb, thyroid stimulating hormone receptor antibody; GREAT, Graves’ Recurrent Events After Therapy; Th17, T helper 17 cells;

Logistic regression analysis was performed using data of 93 patients who consented to treatment with ATDs. In this analysis, the pretreatment PB immunophenotype was the independent variable and presence of high GREAT score (> 2 points) at 24 weeks was the dependent variable. The results showed that activated Th17 cells were significantly associated with high GREAT score (Table 2). Moreover, logistic regression analysis, with the pretreatment PB immunophenotype as the independent variable and TRAb positivity (TRAb ≥ 2.0 U/ml) as the dependent variable, showed that TRAb positivity was associated with the levels of activated Th17 cells (Table 3). Based on these results, we divided the patients into two groups according to the median value of activated Th17 cells, including the activated Th17^high^ group, with activated Th17 cells > 1.67%, and activated Th17^low^ group, with activated Th17 cells ≤ 1.67%. The pretreatment activated Th17^high^ group had a lower percentage of TSH normalization and higher TRAb levels than the activated Th17^low^ group after 24-week treatment (Fig. 5).

**Table 2:** Results of univariate and multivariate logistic regression analyses for prediction of high GREAT score (≥ 2) at 24-weeks of treatment.

		Univariate models		Multivariate model	
		OR	*p*	OR	*p*
CD4^+^ Tcells	Naïve	1.05 (1.01–1.10)	**0.009**	1.06 (0.96–1.20)	0.247
	Central memory	0.95 (0.90–1.00)	0.057	1.04 (0.91–1.20)	0.583
	Effector memory	0.95 (0.89–1.02)	0.161	1.01 (0.87–1.18)	0.907
	TEMRA	1.07 (0.92–1.27)	0.418		
	Activated	1.19 (1.06–1.36)	**0.007**		
CD8^+^ T cells	Naïve	1.01 (0.99–1.05)	0.308		
	Central memory	0.99 (0.94–1.04)	0.714		
	Effector memory	0.98 (0.94–1.01)	0.177	1.00 (0.95–1.05)	0.872
	TEMRA	1.00 (0.97–1.04)	0.834		
	Activated	1.03 (0.95–1.12)	0.430		
CD4^+^ T cell subsets	Th1	1.00 (0.93–1.07)	0.935		
	Activated Th1	1.12 (0.8–1.59)	0.509		
	Th17	0.91 (0.81–1.00)	0.067	0.94 (0.80–1.08)	0.369
	Activated Th17	1.68 (1.11–2.71)	**0.021**	1.68 (1.06–2.88)	**0.041**
	Treg	0.76 (0.53–1.05)	0.106		
	Activated Treg	0.70 (0.37–1.24)	0.230		
	Naive Treg	0.77 (0.33–1.74)	0.531		
	Memory Treg	0.76 (0.52–1.08)	0.137		
	Tfh	0.74 (0.31–1.74)	0.494		
	Activated Tfh	1.22 (0.14–10.8)	0.854		
B cells	Naive	1.03 (0.98–1.07)	0.231		
	IgM memory	0.97 (0.90–1.04)	0.384		
	Central memory	0.94 (0.85–1.02)	0.164	0.96 (0.86–1.06)	0.432
	Double negative	1.01 (0.85–1.20)	0.921		
	Plasmablasts	1.03 (0.90–1.19)	0.669		
Monocytes	Classical	1.01 (0.91–1.12)	0.821		
	Non classical	0.99 (0.89–1.10)	0.849		
Dendritic cells	Myeloid	0.98 (0.95–1.02)	0.367		
	Plasmacytoid	1.02 (0.96–1.10)	0.491		
NK cells	CD16 +	1.03 (0.99–1.08)	0.200		
	CD16-	0.98 (0.92–1.04)	0.526		

Significant values are in bold.

GREAT, graves’ recurrent events after therapy; OR, odds ratio; CI, confidence interval; TEMRA; terminally differentiated effector memory cells, Tfh; follicular helper T cells.

**Table 3:** Results of univariate and multivariate logistic regression analyses for prediction of TRAb positive (≥ 2) at 24 weeks of treatment.

		Univariate models		Multivariate model	
		OR	*p*	OR	*p*
CD4^+^ Tcells	Naïve	1.03 (0.99–1.07)	0.137	0.98 (0.90–1.07)	0.680
	Central memory	0.95 (0.90–1.01)	0.090	0.91 (0.80–1.02)	0.121
	Effector memory	0.99 (0.92–1.07)	0.770		
	TEMRA	0.92 (0.78–1.07)	0.266		
	Activated	1.00 (0.89–1.12)	0.984		
CD8^+^ T cells	Naïve	1.01 (0.98–1.05)	0.393		
	Central memory	0.99 (0.94–1.05)	0.773		
	Effector memory	0.98 (0.94–1.01)	0.195	0.97 (0.93–1.02)	0.270
	TEMRA	1.01 (0.97–1.06)	0.548		
	Activated	1.04 (0.95–1.15)	0.382		
CD4^+^ T cell subsets	Th1	0.99 (0.92–1.07)	0.879		
	Activated Th1	1.08 (0.76–1.60)	0.667		
	Th17	0.97 (0.88–1.07)	0.501		
	Activated Th17	1.79 (1.12–3.13)	**0.027**	1.92 (1.10–3.83)	**0.042**
	Treg	0.78 (0.56–1.08)	0.140		
	Activated Treg	0.66 (0.36–1.20)	0.170		
	Naive Treg	0.60 (0.25–1.42)	0.238	0.23 (0.07–0.68)	0.010
	Memory Treg	0.80 (0.55–1.16)	0.246		
	Tfh	1.41 (0.54–4.07)	0.493		
	Activated Tfh	4.20 (0.35–72.2)	0.286		
B cells	Naive	1.03 (0.99–1.08)	0.160	1.04 (0.99–1.11)	0.147
	IgM memory	0.91 (0.84–0.99)	**0.035**		
	Central memory	0.95 (0.86–1.04)	0.237		
	Double negative	1.24 (1.00–1.62)	0.086	1.32 (1.03–1.78)	**0.045**
	Plasmablasts	1.08 (0.93–1.31)	0.355		
Monocytes	Classical	1.04 (0.94–1.16)	0.439		
	Non classical	0.98 (0.88–1.10)	0.748		
Dendritic cells	Myeloid	1.01 (0.97–1.04)	0.702		
	Plasmacytoid	0.99 (0.92–1.06)	0.670		
NK cells	CD16 +	1.02 (0.98–1.07)	0.325		
	CD16-	0.98 (0.92–1.04)	0.470		

Significant values are in bold.

GREAT, Graves’ Recurrent Events After Therapy; OR, odds ratio; CI, confidence interval; TEMRA; terminally differentiated effector memory cells, Tfh; follicular helper T cells.

Next, we divided the patients into the GREAT score^high^ group (GREAT score ≥ 2) and GREAT score^low^ group (GREAT score < 2). The proportion of patients with normalized thyroid function (0.34 μIU/mL < TSH < 6.5 μIU/mL) after 24 weeks was evaluated in four groups: activated Th17^low^/GREAT score^low^ group (n = 15), activated Th17^high^/GREAT score^low^ group (n = 9), activated Th17^low^/GREAT score^high^ group (n = 30), and activated Th17^high^/GREAT score^high^ group (n = 39). In the pretreatment activated Th17^high^/GREAT score^high^ group, the percentage of normalized thyroid function after 24 weeks was lower and TRAb was higher than in the other three groups (Fig. 5).

The main results of this study are summarized. Compared to healthy subjects, newly onset GD patients had elevated levels of activated Th17 cells, Tfh cells and Th1 cells, and activated Th17 cells were associated with goiter, thyroid function, autoantibodies, and refractory disease. Activated Th17 cells remained unchanged after treatment and were still associated with thyroid gland enlargement after treatment.; activated Tfh cells decreased after treatment, and the decrease correlated with the decrease in thyroid function.

## Discussion

This is the first study to use PB immunophenotyping in treatment-naïve patients with GD to detect immune dysregulation before and after treatment, and to evaluate its relationship with clinical conditions. The main findings at baseline were the high levels of Th17 cells in the patient group than HCs, and that this abnormality was associated with the production of autoantibodies, abnormal thyroid function tests, and goiter. Analysis of data of the patients before and after 24-week treatment showed that the Th17 cell levels at baseline correlated with the GREAT score and post-treatment clinical status, suggesting the potential usefulness of Th17 cells as a prognostic factor in GD. These results suggest the potential role of activated Th17 cells in the clinical findings and intractable pathology. Another important finding of the present study was the high levels of PB Tfh cells and double-negative B cells at baseline, and the correlation between double-negative B cells and TRAb. Treatment with ATDs was associated with reduction in Tfh cells and double-negative B cells as well as correction of thyroid hormone levels.

Th17 cells are a subset of helper T cells known to differentiate via IL-6– and IL-23–dependent activation of STAT3 and TGF-β signaling^15^, and are important in the pathogenesis of various autoimmune diseases^16,17^. In GD patients, highly expressed of Th17^11,18^ cells, RORγt mRNA and CD4^+^IL^−^17^+^ T cells^19^ are reported.; but the present study, for the first time, showed that Th17 cells are associated with goiter. Enlargement of the thyroid gland is closely related to the pathology of GD; TSH receptor-mediated proliferation of thyroid follicular cells, which is mediated through the cAMP-PKA pathway^20^, leads to enlargement of the thyroid gland. We demonstrated in the present study that Th17 cells are associated with goiter, both before and after treatment, when the thyroid function and thyroid autoantibodies improve. This finding suggests that in GD, Th17 cells cause thyroid gland enlargement through a mechanism independent of the activation of TSH receptors by autoantibodies. In autoimmune thyroiditis, there have been some reports evaluating Th17 cells and clinical features, but none have shown the association with thyroid gland enlargement. In newly onset chronic thyroiditis, IL-17 is not associated with thyroid gland enlargement, while the percentage of Th17 cells is strongly associated with TSH^21^. In other words, to examine the effects of Th17 cells and TSHR-independent, it would be necessary to evaluate a population with normal thyroid function or after normalization of thyroid function after treatment. In addition, the levels of Th17 cells remained high despite treatment with ATDs, suggesting that Th17 cells are a central immunological factor in GD that is not affected by thyroid hormones.

IL-17 produced by Th17 cells is known to promote inflammation by inducing various chemokines and cytokines. In this study, we didn’t evaluate cytokines or chemokines, but it is known that Th17 cells and IL-17 are strongly associated in patients with GD^18^. Moreover, in Hashimoto's disease, lymphocytes infiltrate the area surrounding thyroid follicular cells, destroying parenchymal cells, with subsequent infiltration of the inflamed area by Th17 cells^22^. Furthermore, IL-17 is an important regulator of cellular and biological metabolism, and is known to mediate metabolic adjustments that promote proliferation of epithelial cells and skin stem cells^23,24^. IL-17 is also implicated in diseases involving the proliferation of non-immune cells, while IL-17 signaling promotes the proliferation of oligodendrocyte progenitor cells in a model of multiple sclerosis^25^. A study involving fibroblastic reticular cells (FRCs) suggested that FRCs are metabolically reprogrammed by IL-17 to promote glucose uptake that supports cell survival and cell proliferation^26^. Therefore, based on the results of the present and previous studies, we propose that the role of Th17 cells in thyroid follicular cells should be examined from both the destructive aspect associated with inflammation and the cell proliferation aspect related to metabolic regulation. Such investigation will help elucidate the pathogenic process of autoimmune thyroiditis, including both GD and Hashimoto's disease.

Th17 cells are significantly associated with the pathogenesis of intractable GD since they are involved in the pathogenesis of newly onset GD and with the risk of relapse after discontinuation of ATDs. The risk of recurrence after 12–18 months of drug therapy is approximately 50%, and patients who face difficulty in achieving remission with drug therapy are treated with radioiodine therapy or surgery^27^. However, such intractable conditions cannot be predicted at the beginning of the treatment, and there is no effective drug therapy for the treatment of intractable cases. Subpopulations of regulatory B cells have been reported as a prognostic factor for methimazole therapy^28^. The present study showed that Th17 cells are potentially suitable marker for resistance to treatment, and in particular, they can also help in identifying intractable pathologies in combination with the GREAT score. Since Th17 cells are a central immunological factor in GD and are significantly associated with intractable pathology, therapeutic strategies targeting the Th17 immune pathway may be effective in the treatment of intractable cases.

Since the first description of Th17 cells^29^ and their proinflammatory activity, it has become clear that they are involved in the etiology of different autoimmune diseases^30^. Research on Th17 cells and autoimmune thyroiditis (AITD) has examined various aspects, starting with early reports showing that Th17 cells are required for induction in animal models^31^, that GD patients who don’t respond to treatment show elevated levels of IL-17^+^ T cells^11,32^. Similar to the present study, AITD patients have been reported to have elevated levels of Th17 cells in their peripheral blood^18^. And IL-17 levels affected thyrocytes show functional IL-17 receptor expression^33^. Recently, the possible role of different immunoregulatory molecules involved in the induction^34^ or inhibition^35^ of Th17 differentiation has been also reported. In the present study, thyroid functions and Th17 cell activation were associated, and future studies should be recommended. In metabolism aspect, it has been reported that in AITD, enhancement of the glycolytic system of CD4 + T cells is involved in the activation of Th17 cells^36^. Future development of Th17 cell-related therapies, such as antagonists of Th17 cells and therapies involving the glycolytic system of CD4 + T cells, is expected.

In GD, lymphocytes infiltrate the thyroid gland and form a germinal center, followed by production of autoantibodies in this ectopic germinal center^37^. Tfh cells are associated with activation and differentiation of B cells in germinal center, and play a central role in the production of autoantibodies in autoimmune diseases. In the present study, Tfh cells and double-negative B cells were increased in GD patients, and double-negative B cells were associated with TRAb. Moreover, the levels of Tfh cells decreased with reduction in thyroid hormones after treatment. The primary mechanism of action of methimazole and propylthiouracil is inhibition of thyroid hormone production^38,39^, but their effects on the immune system remain unclear. T3 binds to nuclear receptors in the target organs and exerts its biological activity (genomic action)^40^; but, non-genomic actions for T3 and T4 that alter cellular functions independent of transcription have also been described^41^. Thyroid hormones can induce T cell proliferation by binding to TRα and enhance iNOS levels as a genomic effect^42^, and by inducing transcription of TRα through increased expression of PKCζ as non-genomic effect^43^. The association of thyroid hormones with abnormal B cell differentiation and Tfh in the present study suggests that thyroid hormones may act directly on these cells. These results may also explain the immunosuppression caused by ATD administration.

The present study has certain limitations. First, it was a single-center study with a limited sample size, and some patients could not be included in the follow-up analysis due to self-interruption or because they selected surgery or intra-radiation therapy. The immunophenotype used in the study has been standardized by the Human Immunology Project and awaits validation by future multicenter studies. Second, the actual number of PB lymphocytes was also evaluated, but the detection power was low due to the small number of cases that could be evaluated (*n* = 32); and there was no significant decrease in the levels of double-negative B cells after treatment. Third, in this study we did not evaluate Th17 cell function, cellular intracellular expression or transcriptional factors. On the other hand, in this study, we used a standardized flow cytometry assay^44^ by the Human Immunology Project, which we believe is a valid method to assess phenotype.

In conclusion, PB helper T cells and B cells and their differentiation abnormalities were used as PB immunophenotypes in GD. The results of the present study suggest that Th17 cells are associated with the production of autoantibodies, thyroid function tests, goiter and intractable pathology, and may play an important role in GD. Moreover, we demonstrated a direct association between Th17 cells and thyroid gland enlargement, and showed the utility of Th17 cells as prognostic factor. Therapeutic strategies targeting Th17 cells and their immune pathways may be effective, especially in intractable cases. In addition, the decrease in Tfh cells following the fall in thyroid hormone levels suggests the involvement of thyroid hormone itself in the differentiation of Tfh cells and activation of B cells.

## Material and methods

### Patients

A prospective study was conducted involving 117 patients of the GD who visited the outpatient clinic at multiple institutions affiliated to the Hospital of the University of Occupational and Environmental Health, Japan, the key station between June 2015 and December 2018. GD was diagnosed which based on the criteria proposed by the Japanese Thyroid Association^45^. Patients who were pregnant, probably pregnant, or lactating, and those with other autoimmune disease, or malignancy and also those had received previously treatment for GD, were excluded from the study.

### Collection of clinical information

Age, sex, smoking status, presence of Graves’ ophthalmopathy, thyroid gland enlargement, thyroid function tests, autoantibodies, and ultrasound findings were evaluated.

We measured serum levels of fT3 and fT4, TRAb by ECLIA method (ECLusys Reagent TRAb), and TSAb by Bioassay EIA method. Levels of thyroglobulin autoantibody (TgAb) and thyroid peroxidase antibody (TPOAb) were measured using the ECLIA method (ECLusys reagent Anti-Tg / Anti-TPO).

The Graves’ Recurrent Events After Therapy (GREAT) score^46^, a predictive model consisting of clinical and biochemical variables, has been proposed as a tool to predict relapse. The pretreatment GREAT score was calculated (Supplementary Table S9), and used for the assessment of risk of relapse following treatment with ATDs.

### Flow cytometric analysis

For immunophenotyping of PB, peripheral blood mononuclear cells (PBMCs) were isolated from fresh blood samples as described previously^47^ and then analyzed using multicolor cytometry. After staining with antibodies (Supplementary Table S10), the cells were evaluated by multicolored flow cytometry (FACSVerse; BD Biosciences, San Jose, CA) and data were analyzed using FlowJo software (Tree Star, Ashland, OR). The account of the number of mononuclear cells used to perform the cytometric staining was 1.0 × 10^5^. Immune cell subset phenotypes were analyzed using eight color flow cytometry with Tfh cell staining in addition to the standardized protocol of the Human Immunophenotyping Consortium^44^ proposed by the National Institute of Health/Federation of Clinical Immunology Societies. Details of the gating method are shown in Supplementary Fig. S2.

### Study design

We evaluated differences in PB immunophenotypes in 117 patients with newly onset GD and 46 age- and sex-matched healthy controls (HCs). We also analyzed the association of PB immunophenotypes with the clinical findings and intractable pathology in patients with GD. Furthermore, patients received methimazole or propylthiouracil therapy under the clinical care of a thyroid specialist. Based on previous reports^48,49^, the patient was evaluated at 24 weeks. After 24 weeks of therapy, PB immunophenotypes were again assessed to evaluate changes from baseline, relationship with changes in clinical findings, and treatment-resistant cases.

Based on the preliminary data, we estimated a standard deviation of 1.2 for Activated Th17 cells and a difference of 1.3 in detection between HCs and GD group, resulting in the number of subjects required was calculated to be 45 subjects each with the significance level α and power 1-β set as 5% and 80%, respectively.

### Statistics

All values are expressed as median [Q1, Q3]. For comparisons between two groups, the unpaired *t*-test was used when the distribution followed a normal distribution, and the Mann–Whitney test was used when the distribution showed a skewed pattern. The Shapiro–Wilk test was used to evaluate if a continuous variable follows a normal distribution. For categorical data, Fisher's direct probability method was used when the expected value cell was < 5, and the χ^2^ test was used otherwise. Spearman's correlation analysis was performed to examine the relationship between PB immunophenotype and clinical characteristics. The Jonckheere–Terpstra test was used to evaluate the PB immunophenotype for each class of the GREAT classification. Paired-samples *t*-test was used to compare the data at baseline and 24 weeks if the data showed normal distribution, and Wilcoxon's signed rank test when the data showed skewed distribution. Odds ratios for higher GREAT score or higher TRAb at 24 weeks were estimated using univariate and multivariate logistic regression analyses. The Kruskal–Wallis test was used for comparison between the four groups at 24 weeks. Data were analyzed using JMP version 13.0 (SAS Institute Inc., Cary, NC) or SPSS Statistical Software version 25.0 (IBM Corp., Armonk, NY). A *p* value < 0.05 was considered to denote statistically significant difference.

### Ethics statement and clinical trial registration

The study was in accordance with the ethical principles that have their origins in the Declaration of Helsinki and its subsequent amendments. All participants provided written informed consent at the time of study enrolment. All experiments were performed in compliance with relevant Japanese laws and institutional guidelines. The study was approved by the Ethics Committee of the University of Occupational and Environmental Health (Trial registration: #H27-020, date: May 21, 2015). The study was registered on UMIN Clinical Trials Registry (UMIN-CTR) on June 1^st^, 2015 as UMIN000017726.

## Supplementary Information

Supplementary Information.

## Data Availability

Some or all datasets generated during and/or analyzed during the current study are not publicly available but are available from the corresponding author on reasonable request. The data that support the findings of this study are available from the corresponding authors upon request.
