# Supplementary material for: Comprehensive immunophenotypic analysis reveals the pathological involvement of Th17 cells in Graves' disease
Source: Sci Rep. 2022 Oct 7;12:16880. doi: 10.1038/s41598-022-19556-z (PMC9546934; doi:10.1038/s41598-022-19556-z)

Supplementary information

Comprehensive immunophenotypic analysis reveals the pathological involvement of Th17 cells in Graves' disease

Keiichi Torimoto, Yosuke Okada, Shingo Nakayamada, Satoshi Kubo, Akira Kurozumi, Manabu Narisawa, and Yoshiya Tanaka.

First Department of Internal Medicine, School of Medicine, University of Occupational and Environmental Health, Japan, 1-1 Iseigaoka, Yahatanishi-ku, Kitakyushu-shi, 807-8555, Japan

Supplemantary Table S1
Supplementary Table S2
Supplementary Table S3
Supplementary Table S4
Supplementary Table S5
Supplementary Table S6
Supplementary Table S7
Supplementary Table S8
Supplementary Table S9
Supplementary Table S10
Supplementary Table S11
Supplementary Figure S1
Supplementary Figure S2
Supplementary Figure S3

Supplementary Table S1. Differences in lymphocytes, monocytes, dendritic cells, and natural killer (NK) cells between patients with Graves' disease and age- and sex-matched healthy control subjects.

		Healthy control (n=46)	Patients (n=117)	p value
$CD4^+$ Tcells	Naïve	53.9 [38.4, 59.2]	52.4 [40.5, 62.0]	0.860
	Central memory	34.2 [26.6, 41.2]	28.8 [23.9, 36.4]	**0.006**
	Effector memory	10.9 [6.76, 16.0]	11.8 [9.04, 17.3]	0.239
	TEMRA	1.70 [0.94, 2.92]	3.68 [2.87, 4.96]	**<0.001**
	Activated	5.00 [3.59, 6.50]	8.88 [6.37, 11.4]	**<0.001**
$CD8^+$ T cells	Naïve	41.7 [22.6, 61.2]	41.5 [28.1, 54.6]	0.902
	Central memory	5.96 [3.47, 13.0]	4.97 [2.21, 13.3]	0.151
	Effector memory	15.1 [9.14, 28.3]	23.2 [15.1, 30.7]	**0.026**
	TEMRA	27.6 [15.0, 39.9]	23.0 [17.5, 30.8]	0.506
	Activated	7.89 [4.79, 12.9]	12.7 [9.99, 16.3]	**<0.001**
CD4+ T cell subsets	Th1	18.8 [16.3, 24.9]	18.6 [14.7, 23.5]	0.471
	Activated Th1	1.08 [0.70, 1.63]	1.61 [0.95, 2.51]	**<0.001**
	Th17	11.4 [8.19, 14.4]	11.3 [8.77, 14.3]	0.440
	Activated Th17	0.66 [0.47, 0.98]	1.67 [1.14, 2.73]	**<0.001**
	Treg	4.13 [3.47, 5.03]	4.13 [3.47, 5.22]	0.594
	Activated Treg	1.10 [0.89, 1.55]	1.24 [0.83, 1.84]	0.278
	Naïve Treg	0.92 [0.65, 1.50]	0.96 [0.64, 1.42]	0.753
	Memory Treg	3.01 [2.51, 3.59]	3.28 [2.50, 4.26]	0.151
	Tfh	0.72 [0.70, 0.90]	1.06 [0.81, 1.29]	**<0.001**
	Activated Tfh	0.20 [0.13, 0.27]	0.25 [0.16, 0.41]	**0.017**
B cells	Naïve	58.9 [45.4, 66.9]	72.7 [61.7, 78.1]	**<0.001**
	IgM memory	22.1 [18.4, 28.2]	13.7 [10.0, 19.0]	**<0.001**
	Class-swiched	12.8 [7.84, 21.2]	6.15 [3.90, 9.72]	**<0.001**
	IgD^-CD27^- double negative	5.07 [3.80, 6.41]	6.95 [5.47, 8.48]	**<0.001**
	Plasmablast	2.12 [1.36, 3.37]	2.00 [1.08, 3.89]	0.708
Monocytes	Classical	90.1 [83.8, 92.2]	89.2 [85.7, 91.7]	0.835
	Non classical	9.22 [6.52, 14.5]	8.07 [6.38, 12.2]	0.472
Dendritic cells	Myeloid	83.4 [78.3, 86.8]	78.7 [70.0, 85.1]	**0.005**
	Plasmacytoid	5.73 [4.21, 6.86]	7.41 [5.07, 12.7]	**<0.001**
NK cells	$CD16^+$	95.4 [92.3, 97.3]	90.2 [82.7, 93.5]	**<0.001**
	$CD16^-$	4.24 [2.32, 5.15]	4.76 [3.19, 7.84]	0.100

Data are median [Q1, Q3] percentage. P values by Student's t-test or Mann-Whitney U-test. See Table 2 for abbreviations.

Supplementary Table S2. Correlations between clinical features and peripheral cell phenotype at baseline.

			TRAb	TSAb	fT3	fT4	Rt. lobe	Lt. lobe	Isthmus
CD4$^+$ Tcells	Naïve	r	**0.221**	0.035	**0.254**	**0.302**	0.118	0.080	**0.210**
		p value	0.017	0.718	0.006	0.001	0.205	0.392	0.024
	Central memory	r	-0.178	0	**-0.190**	**-0.218**	0.051	0.034	-0.178
		p value	0.054	1.000	0.042	0.018	0.584	0.712	0.057
	Effector memory	r	**-0.253**	-0.091	**-0.216**	**-0.184**	-0.173	-0.100	**-0.207**
		p value	0.006	0.348	0.020	0.047	0.062	0.281	0.026
	TEMRA	r	-0.013	-0.125	-0.093	**-0.189**	-0.103	0.001	0.060
		p value	0.891	0.199	0.318	0.041	0.271	0.988	0.522
	Activated	r	0.085	0.099	0.144	0.126	0.116	0.155	-0.047
		p value	0.365	0.306	0.123	0.174	0.214	0.095	0.622
CD8$^+$ T cells	Naïve	r	0.010	-0.018	0.074	0.107	0.021	-0.064	0.128
		p value	0.919	0.857	0.435	0.254	0.821	0.495	0.175
	Central memory	r	-0.127	-0.034	-0.035	-0.144	-0.007	0.055	-0.09
		p value	0.174	0.728	0.707	0.122	0.941	0.557	0.340
	Effector memory	r	-0.080	0.015	-0.060	-0.018	0.049	0.083	-0.088
		p value	0.392	0.876	0.527	0.846	0.601	0.375	0.351
	TEMRA	r	0.116	-0.058	-0.010	-0.023	-0.041	-0.034	-0.019
		p value	0.217	0.551	0.912	0.804	0.666	0.719	0.841
	Activated	r	0.010	-0.013	0.154	0.129	0.142	0.117	-0.052
		p value	0.913	0.897	0.100	0.169	0.128	0.210	0.586
CD4+ T cell subsets	Th1	r	-0.025	-0.086	-0.170	**-0.187**	-0.141	-0.096	-0.123
		p value	0.788	0.376	0.069	0.043	0.129	0.302	0.192
	Activated Th1	r	0.141	0.066	0.163	**0.199**	0.091	**0.195**	0.063
		p value	0.128	0.499	0.081	0.032	0.329	0.036	0.507
	Th17	r	-0.111	0.017	-0.108	**-0.182**	-0.018	-0.083	**-0.262**
		p value	0.233	0.862	0.250	0.049	0.847	0.372	0.005
	Activated Th17	r	**0.212**	0.077	**0.288**	**0.253**	**0.320**	**0.292**	0.138
		p value	0.022	0.431	0.002	0.006	0.000	0.001	0.141
	Treg	r	-0.113	-0.033	-0.025	-0.088	-0.008	-0.048	-0.102
		p value	0.225	0.731	0.791	0.346	0.933	0.607	0.277
	Activated Treg	r	0.047	0.051	0.087	0.061	0.061	0.029	-0.073
		p value	0.619	0.604	0.356	0.513	0.514	0.752	0.437
	Naïve Treg	r	0.094	0.094	0.168	0.095	0.109	0.016	0.143
		p value	0.313	0.336	0.072	0.311	0.241	0.864	0.129
	Memory Treg	r	-0.145	-0.061	-0.116	-0.155	-0.052	-0.073	-0.154
		p value	0.118	0.530	0.213	0.096	0.581	0.436	0.100
	Tfh	r	-0.075	0.002	0.003	0.007	-0.043	-0.002	0.050
		p value	0.427	0.984	0.971	0.943	0.650	0.986	0.602
	Activated Tfh	r	-0.046	0.017	-0.003	0.065	-0.087	-0.067	-0.051
		p value	0.629	0.860	0.975	0.488	0.355	0.479	0.593
B cells	Naïve	r	**0.240**	0.123	0.245	0.251	**0.203**	0.116	0.174
		p value	0.010	0.211	0.008	0.007	0.029	0.217	0.065
	IgM memory	r	**-0.337**	-0.165	**-0.290**	**-0.337**	**-0.229**	-0.123	-0.127
		p value	<0.001	0.090	0.002	<0.001	0.014	0.191	0.179
	Class-swiched	r	**-0.233**	-0.126	-0.177	-0.146	-0.171	-0.078	**-0.206**
		p value	0.012	0.199	0.060	0.120	0.068	0.407	0.029
	IgD$^-$CD27$^-$ double negative	r	**0.277**	**0.226**	0.087	0.107	-0.027	0.057	0.039
		p value	0.003	0.020	0.358	0.255	0.771	0.548	0.680
	Plasmablast	r	0.025	0.094	0.060	0.052	-0.134	-0.044	-0.061
		p value	0.795	0.338	0.529	0.583	0.153	0.640	0.521
Monocytes	Classical	r	0.052	**-0.239**	-0.012	0.017	0.038	-0.034	-0.014
		p value	0.583	0.014	0.895	0.858	0.683	0.721	0.886
	Non classical	r	-0.041	**0.234**	-0.002	-0.036	-0.017	0.054	0.003
		p value	0.665	0.016	0.983	0.706	0.858	0.568	0.971
Dendritic cells	Myeloid	r	0.040	0.088	0.136	0.123	-0.071	0.034	0.018
		p value	0.672	0.369	0.149	0.189	0.452	0.716	0.85
	Plasmacytoid	r	-0.007	-0.067	-0.029	-0.037	-0.004	-0.07	0.109
		p value	0.938	0.495	0.756	0.692	0.965	0.456	0.248
NK cells	CD16$^+$	r	-0.049	-0.082	-0.124	-0.093	-0.016	0.012	-0.028
		p value	0.603	0.401	0.190	0.322	0.864	0.895	0.768
	CD16$^-$	r	0.141	0.151	**0.251**	**0.244**	-0.008	-0.016	0.112
		p value	0.132	0.122	0.007	0.009	0.936	0.867	0.239

Data are results of Spearman’s correlation analysis.See Tables 1 and 2 for abbreviations.

Supplementary Table S3. Effects of 24-week treatment with antithyroid drugs on various clinical parameters.

	baseline	24 weeks	p value
Free triiodothyronine (fT3), pg/mL	12.1 [6.97, 22.0]	3.02 [2.71, 3.68]	**<0.001**
Free thyroxine (fT4), ng/dL	4.12 [2.82, 7.21]	1.20 [1.06, 1.44]	**<0.001**
TRAb, IU/L	9.00 [4.00, 19.6]	3.60 [1.60, 7.40]	**<0.001**
TSAb, %	522.0 [233.5, 1299.0]	217.0 [144.5, 574.5]	**<0.001**
Right lobe size on ultrasoud, mm	16.0 [14.0, 18.1]	16.0 [14.0, 19.0]	0.853
Left lobe size on ultrasoud, mm	16.0 [13.3, 18.0]	16.7 [13.0, 18.0]	0.642
Isthmus size on ultrasound, mm	3.00 [2.00, 4.00]	2.70 [2.00, 3.70]	0.853
Velocity in the right STA, cm/s	65.0 [45.9, 87.8]	51.5 [34.9, 66.5]	**<0.001**
Velocity in the left STA, cm/s	63.6 [47.0, 87.0]	51.0 [34.0, 65.0]	**<0.001**
GREAT score	3.00 [1.00, 4.00]	2.00 [1.00, 3.00]	**<0.001**

Data are median [Q1, Q3]. P values by the paired-samples t-test or with Wilcoxon's signed rank test.
See Table 1 for abbreviations.

Supplementary Table S4. Effects of 24-week treatment with antithyroid drugs on the proportions of immune cells in peripheral blood.

		baseline	24 weeks	p value
$CD4^+$ T cells	Naive	52.5 [40.5, 61.9]	55.7 [46.2, 61.8]	**<0.001**
	Central memory	28.0 [23.0, 36.2]	30.0 [24.8, 36.2]	0.132
	Effector memory	12.8 [10.0, 19.0]	9.86 [7.53, 12.9]	**<0.001**
	TEMRA	3.56 [2.77, 4.76]	3.39 [2.65, 4.81]	0.914
	Activated	9.72 [6.74, 12.5]	7 [5.73, 10.1]	**<0.001**
$CD8^+$ T cells	Naive	39.9 [25.9, 53.2]	47.9 [34.8, 55.3]	**<0.001**
	Central memory	4.66 [1.89, 13.7]	6.05 [2.27, 14.6]	0.253
	Effector memory	24.2 [15.4, 32.2]	19.3 [13.8, 27.7]	**0.030**
	TEMRA	23.2 [16.8, 32.4]	21.4 [15.7, 30.5]	0.183
	Activated	13.4 [9.94, 17.0]	10.9 [8.19, 15.2]	**<0.001**
$CD4^+$ T cell subsets	Th1	19.5 [14.7, 24.3]	18.3 [15.8, 22.7]	0.133
	Activated Th1	1.76 [1.05, 2.77]	1.49 [1.01, 2.02]	**0.017**
	Th17	11.5 [8.73, 14.4]	10.8 [8.08, 14.3]	**0.007**
	Activated Th17	1.78 [1.20, 2.81]	1.59 [1.16, 2.43]	0.215
	Treg	4.02 [3.52, 5.23]	4.4 [3.84, 5.06]	**0.046**
	Activated Treg	1.25 [0.86, 1.85]	1.17 [0.89, 1.49]	0.082
	Naive Treg	0.89 [0.57, 1.23]	1.1 [0.7, 1.56]	**<0.001**
	Memory Treg	3.26 [2.53, 4.3]	3.19 [2.63, 4.26]	0.931
	Tfh	1.06 [0.86, 1.28]	0.8 [0.65, 1.16]	**<0.001**
	Activated Tfh	0.28 [0.18, 0.43]	0.17 [0.11, 0.33]	**<0.001**
B cells	Naive	72.7 [63.0, 78.3]	71.4 [61.9, 78.4]	0.190
	IgM memory	13.7 [10.2, 17.6]	14.8 [10.3, 19.9]	**0.007**
	Central memory	5.98 [4.11, 10.7]	7.45 [4.39, 11.8]	**0.003**
	IgD^-CD27^- double negative	6.85 [5.53, 8.6]	5.92 [4.74, 7.53]	**<0.001**
	Plasmablasts	1.99 [1.10, 4.12]	1.19 [0.49, 2.16]	**<0.001**
Monocytes	Classical	89.0 [85.6, 91.5]	92.1 [88.4, 93.8]	**0.006**
	Non classical	9.18 [6.88, 12.7]	6.53 [4.69, 9.42]	**0.001**
Dendritic cells	Myeloid	79.2 [68.1, 85.2]	78.1 [69.4, 82.0]	0.311
	Plasmacytoid	7.10 [4.75, 13.25]	7.93 [5.23, 11.9]	0.992
NK cells	CD16+	91.4 [85.0, 93.8]	90.7 [84.2, 94.1]	0.489
	CD16-	4.46 [2.91, 6.58]	4.2 [2.45, 6.45]	0.095

Data are mean±SD percentage. P values by the paired-samples t-test or with Wilcoxon's signed rank test.

See Table 2 for abbreviations.

Supplementary Table S5. Effects of 24-week treatment with antithyroid drugs on numbers of immune cells in peripheral blood.

		baseline (n=32)	24 weeks (n=32)	p value
$CD4^+$ T cells	Naive	357.3 [257.3, 492.7]	453.2 [329.5, 595.9]	**<0.001**
	Central memory	207.5 [156, 266.4]	236.4 [170.7, 331.6]	0.096
	Effector memory	96.6 [64.5, 130.8]	79.7 [59.3, 107.4]	**0.046**
	TEMRA	24.5 [17.3, 38.7]	27.3 [19.5, 43.7]	0.109
	Activated	72.1 [45.9, 99.1]	57.1 [42.9, 81.9]	0.139
$CD8^+$ T cells	Naive	112.8 [68.2, 171.2]	161.1 [84.2, 224.8]	**0.008**
	Central memory	16.8 [6, 34.3]	18.5 [7.4, 48.1]	0.695
	Effector memory	74.2 [48.7, 95.8]	66.4 [36.1, 90.4]	0.487
	TEMRA	69.1 [40.9, 113.5]	62.6 [39.8, 118.1]	0.297
	Activated	40.1 [27.1, 59.4]	35.5 [24.2, 54.2]	0.414
$CD4^+$ T cell subsets	Th1	141.6 [89.1, 181.6]	150.3 [107.9, 215.9]	0.605
	Activated Th1	11.2 [7.10, 20.9]	10.8 [8.10, 16.4]	0.478
	Th17	86.1 [60.4, 112.9]	88 [58.0, 117.4]	0.466
	Activated Th17	12.4 [8.10, 21.4]	12.2 [8.90, 23.2]	0.387
	Treg	30.9 [20.6, 40.7]	38.2 [27.3, 49.4]	**0.001**
	Activated Treg	9.53 [6.64, 14.14]	9.30 [7.08, 12.73]	0.514
	Naive Treg	5.87 [3.67, 9.92]	8.67 [5.23, 15.25]	**<0.001**
	Memory Treg	23.1 [17.0, 30.5]	25.7 [19.7, 33.2]	0.128
	Tfh	7.20 [5.35, 9.93]	6.64 [4.87, 9.73]	**0.018**
	Activated Tfh	2.09 [1.33, 2.92]	1.53 [0.86, 2.86]	**0.046**
B cells	Naive	60.7 [35.7, 88.0]	54.6 [31.4, 100.9]	0.594
	IgM memory	10.6 [6.60, 16.2]	12.77 [7.89, 17.82]	0.526
	Central memory	4.75 [2.69, 9.1]	6.44 [3.49, 9.31]	0.526
	IgD^-CD27^- double negative	5.99 [3.66, 8.81]	4.84 [3.58, 7.63]	0.235
	Plasmablasts	1.67 [0.91, 3.32]	1.09 [0.48, 1.67]	**0.003**
Monocytes	Classical	337.5 [284.5, 432]	301.7 [243.1, 370.5]	**0.020**
	Non classical	36.4 [25.0, 52.3]	22.1 [13.1, 34.2]	**0.022**
Dendritic cells	Myeloid	61.8 [35.5, 92.7]	36.3 [24.1, 57.5]	0.026
	Plasmacytoid	5.58 [3.57, 8.59]	3.81 [2.94, 5.69]	0.031
NK cells	CD16+	402.1 [160.9, 624.0]	293.5 [161.6, 578.5]	0.710
	CD16-	17.4 [11.0, 21.7]	12.48 [7.17, 22.49]	0.162

Data are mean±SD percentage. P values by the paired-samples t-test or with Wilcoxon's signed rank test.

See Table 2 for abbreviations.

Supplementary Table S6. Correlations between changes of clinical features and changes of the proprtions of peripheral immune cells following treatment of Graves' disease.

			ΔTRAb	ΔTSAb	ΔfT3	ΔfT4	ΔRt. lobe	ΔLt. lobe	ΔIsthmus
$CD4^+$ Tcells	ΔNaïve	r	0.028	-0.069	0.169	0.133	**-0.285**	-0.221	-0.195
		p value	0.811	0.589	0.153	0.258	0.016	0.064	0.104
	ΔCentral memory	r	-0.023	-0.012	0.021	0.051	0.138	0.061	-0.015
		p value	0.848	0.923	0.859	0.666	0.251	0.611	0.899
	ΔEffector memory	r	0.083	0.199	-0.073	-0.065	0.065	0.067	0.129
		p value	0.482	0.117	0.538	0.584	0.593	0.580	0.282
	ΔTEMRA	r	-0.021	-0.198	-0.143	-0.193	0.106	0.080	0.038
		p value	0.857	0.120	0.228	0.100	0.379	0.509	0.753
	ΔActivated	r	0.095	0.046	0.227	0.197	-0.190	-0.130	-0.076
		p value	0.421	0.723	0.054	0.093	0.113	0.282	0.527
$CD8^+$ T cells	ΔNaïve	r	-0.169	0.050	0.104	0.103	-0.229	-0.084	-0.063
		p value	0.156	0.701	0.39	0.387	0.059	0.495	0.607
	ΔCentral memory	r	-0.042	-0.117	-0.07	-0.065	-0.117	0.001	0.061
		p value	0.725	0.364	0.565	0.586	0.340	0.998	0.621
	ΔEffector memory	r	0.069	0.202	-0.064	-0.007	0.232	0.152	0.158
		p value	0.565	0.116	0.598	0.957	0.055	0.213	0.196
	ΔTEMRA	r	0.020	-0.130	0.006	-0.054	0.069	-0.057	-0.054
		p value	0.867	0.312	0.961	0.651	0.572	0.645	0.662
	ΔActivated	r	0.082	0.035	**0.347**	**0.268**	-0.210	**-0.272**	-0.157
		p value	0.495	0.787	0.003	0.023	0.083	0.024	0.197
CD4+ T cell subsets	ΔTh1	r	-0.094	-0.025	-0.175	-0.071	-0.087	-0.06	-0.159
		p value	0.427	0.849	0.138	0.547	0.470	0.620	0.185
	ΔActivated Th1	r	0.131	0.223	0.219	**0.261**	-0.024	0.005	-0.159
		p value	0.267	0.079	0.062	0.025	0.840	0.968	0.186
	ΔTh17	r	0.065	0.039	0.087	0.015	0.132	0.078	0.227
		p value	0.582	0.761	0.467	0.901	0.273	0.520	0.057
	ΔActivated Th17	r	0.179	0.157	0.131	0.070	-0.021	0.008	-0.066
		p value	0.128	0.221	0.268	0.555	0.862	0.949	0.587
	ΔTreg	r	0.123	-0.148	0.184	0.171	0.099	-0.069	0.048
		p value	0.299	0.250	0.121	0.147	0.416	0.569	0.693
	ΔActivated Treg	r	-0.013	0.045	0.184	0.109	0.020	0.047	-0.056
		p value	0.915	0.728	0.123	0.360	0.869	0.702	0.643
	ΔNaïve Treg	r	-0.188	-0.085	-0.134	-0.138	0.122	0.037	0.121
		p value	0.110	0.513	0.262	0.245	0.313	0.762	0.319
	ΔMemory Treg	r	-0.069	-0.120	0.124	0.015	0.049	-0.100	-0.046
		p value	0.563	0.352	0.298	0.903	0.689	0.411	0.707
	ΔTfh	r	-0.017	-0.029	-0.046	-0.040	-0.007	0.138	-0.040
		p value	0.877	0.812	0.684	0.725	0.955	0.226	0.726
	ΔActivated Tfh	r	0.001	0.095	**0.260**	**0.234**	-0.143	-0.038	0.130
		p value	0.991	0.461	0.028	0.047	0.234	0.752	0.282
B cells	ΔNaïve	r	-0.044	0.158	0.084	0.181	0.027	0.020	**-0.248**
		p value	0.716	0.224	0.482	0.129	0.825	0.871	0.040
	ΔIgM memory	r	-0.090	**-0.314**	**-0.245**	**-0.277**	-0.168	-0.103	0.231
		p value	0.451	0.014	0.038	0.018	0.168	0.398	0.057
	ΔClass-swiched	r	-0.028	-0.074	0.067	0.041	0.088	0.046	0.166
		p value	0.817	0.570	0.575	0.731	0.473	0.708	0.173
	Δ IgD^-CD27^- double negative	r	**0.282**	0.198	0.118	0.049	0.032	0.015	0.155
		p value	0.017	0.126	0.325	0.684	0.793	0.900	0.203
	ΔPlasmablast	r	-0.043	-0.089	0.128	0.016	**-0.249**	-0.136	-0.223
		p value	0.721	0.494	0.285	0.896	0.039	0.267	0.065
Monocytes	ΔClassical	r	-0.16	**-0.257**	**-0.271**	-0.225	0.222	-0.126	0.099
		p value	0.187	0.048	0.024	0.061	0.071	0.309	0.425
	ΔNon classical	r	0.164	0.214	**0.305**	0.217	-0.235	0.044	-0.152
		p value	0.175	0.101	0.010	0.072	0.055	0.724	0.221
Dendritic cells	ΔMyeloid	r	-0.119	0.143	0.213	0.187	-0.110	-0.176	-0.076
		p value	0.327	0.275	0.077	0.121	0.376	0.154	0.540
	ΔPlasmacytoid	r	0.061	-0.153	-0.190	-0.183	0.124	0.007	0.026
		p value	0.618	0.244	0.115	0.129	0.318	0.955	0.836
NK cells	$\Delta CD16^+$	r	-0.139	-0.071	-0.153	-0.174	0.053	-0.059	0.195
		p value	0.252	0.591	0.207	0.150	0.671	0.634	0.113
	$\Delta CD16^-$	r	0.075	-0.007	0.230	0.212	-0.021	-0.086	-0.216
		p value	0.535	0.959	0.055	0.078	0.869	0.492	0.079

Data are results of Spearman’s correlation analysis.

See Tables 1 and 2 for abbreviations.

Supplementary Table S7. Correlations between changes of clinical features and changes of the numbers of peripheral immune cells following 24-week treatment of Graves' disease.

			ΔTRAb	ΔTSAb	ΔfT3	ΔfT4	ΔRt. lobe	ΔLt. lobe	ΔIsthmus
$CD4^+$ Tcells	ΔNaïve	r	-0.114	-0.105	-0.120	-0.053	**-0.293**	**-0.274**	-0.173
		p value	0.340	0.422	0.319	0.659	0.015	0.023	0.155
	ΔCentral memory	r	-0.113	-0.127	-0.104	-0.027	-0.017	-0.181	-0.159
		p value	0.345	0.330	0.390	0.820	0.888	0.137	0.193
	ΔEffector memory	r	0.055	0.132	-0.15	-0.102	0.040	-0.021	0.086
		p value	0.646	0.312	0.212	0.394	0.744	0.864	0.483
	ΔTEMRA	r	-0.123	-0.251	-0.220	**-0.277**	0.045	0.050	0.026
		p value	0.302	0.051	0.065	0.018	0.716	0.682	0.833
	ΔActivated	r	0.013	0.025	0.204	0.184	-0.178	-0.126	-0.058
		p value	0.912	0.846	0.088	0.121	0.144	0.304	0.639
$CD8^+$ T cells	ΔNaïve	r	-0.162	-0.090	-0.102	-0.003	**-0.247**	-0.119	-0.056
		p value	0.181	0.494	0.405	0.980	0.044	0.338	0.653
	ΔCentral memory	r	-0.083	-0.121	-0.067	0.007	-0.105	0.001	0.032
		p value	0.493	0.357	0.584	0.955	0.400	0.997	0.798
	ΔEffector memory	r	0.068	0.094	-0.080	0.024	0.133	0.086	0.111
		p value	0.574	0.475	0.516	0.847	0.283	0.490	0.371
	ΔTEMRA	r	-0.010	-0.261	-0.120	-0.143	-0.031	-0.152	-0.088
		p value	0.936	0.044	0.325	0.237	0.802	0.220	0.479
	ΔActivated	r	0.022	-0.043	**0.281**	**0.273**	-0.155	-0.152	-0.109
		p value	0.857	0.747	0.019	0.023	0.212	0.218	0.379
CD4+ T cell subsets	ΔTh1	r	-0.088	-0.111	-0.131	-0.015	-0.104	-0.178	-0.230
		p value	0.462	0.396	0.276	0.900	0.394	0.143	0.058
	ΔActivated Th1	r	0.048	0.133	0.167	**0.246**	-0.074	-0.062	-0.150
		p value	0.691	0.308	0.164	0.037	0.546	0.612	0.220
	ΔTh17	r	-0.004	-0.010	-0.039	-0.037	0.019	-0.121	0.067
		p value	0.976	0.941	0.747	0.759	0.879	0.325	0.589
	ΔActivated Th17	r	0.074	0.160	0.048	0.021	0.001	0.028	-0.016
		p value	0.540	0.222	0.694	0.861	0.994	0.821	0.897
	ΔTreg	r	-0.181	-0.115	-0.046	-0.042	-0.001	-0.201	-0.101
		p value	0.131	0.380	0.708	0.729	0.991	0.100	0.412
	ΔActivated Treg	r	-0.104	-0.003	0.154	0.122	0.033	-0.009	-0.123
		p value	0.388	0.982	0.203	0.313	0.791	0.944	0.316
	ΔNaïve Treg	r	-0.214	-0.168	-0.212	-0.150	0.004	-0.097	0.066
		p value	0.073	0.199	0.079	0.213	0.971	0.432	0.595
	ΔMemory Treg	r	-0.162	-0.054	0.086	0.045	-0.011	-0.221	-0.125
		p value	0.179	0.681	0.481	0.713	0.930	0.070	0.309
	ΔTfh	r	-0.093	-0.017	0.101	0.070	-0.009	-0.085	0.019
		p value	0.441	0.898	0.406	0.563	0.942	0.490	0.877
	ΔActivated Tfh	r	-0.047	0.033	**0.288**	**0.280**	-0.171	-0.091	0.051
		p value	0.700	0.801	0.016	0.018	0.161	0.456	0.678
B cells	ΔNaïve	r	0.096	0.165	0.016	0.077	0.046	0.107	-0.078
		p value	0.428	0.212	0.898	0.525	0.713	0.390	0.532
	ΔIgM memory	r	0.017	-0.039	-0.060	-0.015	-0.102	0.013	0.065
		p value	0.888	0.771	0.619	0.904	0.414	0.919	0.602
	ΔClass-swiched	r	0.096	0.065	0.083	0.119	0.170	0.152	0.084
		p value	0.432	0.626	0.497	0.328	0.170	0.220	0.502
	ΔIgD^-CD27^- double negative	r	0.229	0.175	0.113	0.118	0.138	0.181	0.142
		p value	0.057	0.184	0.351	0.331	0.265	0.142	0.250
	ΔPlasmablast	r	0.013	-0.002	0.209	0.167	-0.115	-0.048	-0.119
		p value	0.915	0.987	0.083	0.166	0.354	0.698	0.338
Monocytes	ΔClassical	r	0.116	0.039	**0.249**	0.215	0.077	-0.009	-0.076
		p value	0.338	0.767	0.039	0.074	0.533	0.942	0.540
	ΔNon classical	r	0.179	**0.257**	**0.372**	**0.276**	-0.206	-0.006	**-0.249**
		p value	0.138	0.048	0.002	0.021	0.095	0.962	0.042
Dendritic cells	ΔMyeloid	r	0.033	0.216	**0.262**	0.128	-0.002	-0.001	-0.059
		p value	0.786	0.097	0.030	0.293	0.986	0.994	0.637
	ΔPlasmacytoid	r	0.020	0.102	0.037	-0.051	0.062	0.026	0.084
		p value	0.872	0.438	0.762	0.677	0.617	0.832	0.500
NK cells	Δ$CD16^+$	r	0.045	0.061	-0.083	-0.107	-0.136	-0.053	0.016
		p value	0.714	0.645	0.500	0.378	0.272	0.668	0.899
	Δ$CD16^-$	r	0.234	0.194	0.202	0.116	-0.189	-0.123	-0.224
		p value	0.051	0.137	0.097	0.338	0.125	0.323	0.069

Data are results of Spearman’s correlation analysis.

See Tables 1 and 2 for abbreviations.

Supplementary Table S8. Correlations between clinical features and peripheral cell phenotype following 24-week treatment of Graves' disease.

			TRAb	TSAb	fT3	fT4	Rt. lobe	Lt. lobe	Isthmus
$CD4^+$ T cells	Naïve	r	0.073	-0.026	0.006	-0.013	0.075	0.064	0.181
		p value	0.539	0.837	0.957	0.916	0.534	0.595	0.135
	Central memory	r	-0.160	-0.005	-0.096	-0.106	-0.049	-0.029	-0.211
		p value	0.172	0.969	0.416	0.368	0.688	0.810	0.080
	Effector memory	r	-0.131	-0.045	0.087	-0.053	-0.008	-0.098	-0.188
		p value	0.268	0.719	0.462	0.653	0.948	0.417	0.119
	TEMRA	r	0.041	-0.031	0.061	-0.008	0.021	0.139	0.146
		p value	0.730	0.808	0.606	0.944	0.861	0.247	0.227
	Activated	r	-0.149	-0.097	-0.085	**-0.267**	-0.006	0.118	-0.066
		p value	0.206	0.443	0.474	0.022	0.961	0.327	0.589
$CD8^+$ T cells	Naïve	r	-0.020	-0.039	-0.186	-0.105	0.011	0.001	**0.247**
		p value	0.868	0.762	0.117	0.378	0.928	0.998	0.043
	Central memory	r	-0.131	-0.035	-0.024	-0.075	-0.062	0.038	-0.201
		p value	0.271	0.788	0.844	0.533	0.610	0.759	0.100
	Effector memory	r	-0.031	-0.073	0.158	0.210	0.098	0.092	-0.008
		p value	0.799	0.567	0.185	0.077	0.425	0.452	0.946
	TEMRA	r	0.168	0.039	0.180	0.137	0.027	0.068	0.019
		p value	0.157	0.764	0.131	0.251	0.826	0.577	0.880
	Activated	r	0.038	0.057	0.041	-0.133	-0.032	-0.031	-0.187
		p value	0.751	0.660	0.736	0.266	0.797	0.804	0.126
CD4+ T cell subsets	Th1	r	-0.031	-0.022	0.054	0.085	-0.180	-0.130	-0.115
		p value	0.793	0.863	0.648	0.473	0.133	0.279	0.345
	Activated Th1	r	-0.094	-0.067	0.054	0.006	-0.138	-0.099	-0.009
		p value	0.424	0.599	0.647	0.958	0.252	0.411	0.939
	Th17	r	-0.059	0.102	-0.046	-0.103	0.035	-0.014	-0.147
		p value	0.619	0.420	0.700	0.381	0.773	0.909	0.224
	Activated Th17	r	0.039	0.081	0.057	-0.188	**0.277**	**0.268**	0.202
		p value	0.745	0.522	0.632	0.108	0.019	0.024	0.093
	Treg	r	-0.076	-0.065	0.017	-0.097	-0.048	-0.115	-0.190
		p value	0.525	0.610	0.887	0.416	0.691	0.344	0.118
	Activated Treg	r	0.035	0.011	0.036	-0.022	-0.051	-0.037	-0.121
		p value	0.770	0.929	0.760	0.856	0.677	0.764	0.322
	Naïve Treg	r	0.023	-0.020	-0.051	-0.097	0.133	0.033	0.201
		p value	0.846	0.874	0.671	0.417	0.271	0.787	0.098
	Memory Treg	r	-0.112	-0.074	0.034	0.003	-0.105	-0.118	**-0.251**
		p value	0.347	0.563	0.775	0.977	0.389	0.330	0.037
	Tfh	r	-0.145	**-0.256**	0.065	0.162	-0.009	-0.017	0.034
		p value	0.219	0.040	0.580	0.169	0.938	0.888	0.779
	Activated Tfh	r	-0.083	-0.198	-0.221	-0.111	-0.213	-0.181	-0.220
		p value	0.484	0.114	0.059	0.346	0.075	0.130	0.068
B cells	Naïve	r	0.201	0.055	0.007	0.035	0.057	-0.028	0.097
		p value	0.088	0.666	0.953	0.771	0.642	0.821	0.427
	IgM memory	r	-0.192	-0.024	-0.003	-0.027	-0.004	0.044	-0.015
		p value	0.104	0.853	0.983	0.822	0.976	0.715	0.903
	Class-swiched	r	**-0.266**	-0.170	-0.033	-0.029	-0.152	-0.063	-0.213
		p value	0.023	0.180	0.784	0.809	0.208	0.603	0.079
	IgD^-CD27^- double negative	r	0.041	0.048	0.137	0.065	0.027	0.092	0.055
		p value	0.732	0.706	0.247	0.588	0.824	0.450	0.654
	Plasmablast	r	-0.123	0.020	-0.011	0.067	0.062	0.088	0.137
		p value	0.301	0.876	0.924	0.572	0.609	0.469	0.260
Monocytes	Classical	r	0.136	0.033	0.047	-0.043	0.197	0.057	**0.265**
		p value	0.259	0.797	0.698	0.721	0.108	0.643	0.030
	Non classical	r	-0.071	0.119	0.036	0.052	-0.119	0.018	-0.213
		p value	0.555	0.356	0.765	0.668	0.336	0.883	0.084
Dendritic cells	Myeloid	r	0.045	-0.168	0.190	0.140	-0.02	0.043	-0.100
		p value	0.708	0.192	0.112	0.245	0.870	0.730	0.421
	Plasmacytoid	r	-0.202	0.020	-0.029	-0.175	0.139	0.011	0.101
		p value	0.091	0.878	0.810	0.145	0.259	0.928	0.416
NK cells	$CD16^+$	r	-0.122	-0.061	0.027	0.159	-0.091	-0.127	-0.173
		p value	0.310	0.636	0.827	0.187	0.459	0.304	0.161
	$CD16^-$	r	0.079	0.040	-0.031	-0.151	0.057	0.023	0.13
		p value	0.514	0.759	0.795	0.208	0.643	0.853	0.295

Data are results of Spearman’s correlation analysis.

See Tables 1 and 2 for abbreviations.

Supplementary Table S9. GREAT score for the outcome of therapy with antithyroid drugs in Graves' disease.

Marker	GREAT score	Recurrence risk
Age, years		
≥40	0	
<40	1	
Serum free thyroxine, ng/dL		
<3.1	0	
≥3.1	1	
Serum TRAb, IU/L		
<6	0	
6-19.9	1	
≥20	2	
Goiter size[a]		
Grade 0- Ⅰ	0	
Grade Ⅱ-Ⅲ	2	
Risk stratification	GREAT score 0-1=class Ⅰ	Recurrences 16%
	GREAT score 2-3=class Ⅱ	Recurrences 44%
	GREAT score 4-6=class Ⅲ	Recurrences 68%

GREAT, Graves' Recurrent Events After Therapy; TRAb, thyroid stimulating hormone receptor antibody

[a]World Health Organization grade 0=thyroid not or distinctly palpable; grade I=thyroid easily palpable and visible with head in normal or raised position; grade II=thyroid easily visible with head in normal position; grade III=goitre visible at a distance.

Supplementary Table S10. Eight-colour antibody panels used in the study

	T cells	Treg	Th1, Th2, Th17	Tfh	B cells	DCs, monocytes, NK cells
FITC	Live or dead	Live or dead	Live or dead	Live or dead	Live or dead	Live or dead
PE	CCR7	CD25	CXCR3	CXCR5	CD24	CD56
PerCP-Cy5.5	CD4	CD4	CD4	CXCR3	CD19	CD123
PE-Cy7	CD45RA	CCR4	CCR6	CCR6	CD27	CD11c
APC	CD38	CD127	CD38	ICOS	CD38	CD16
APC-H7	CD8	CD45RO	CD8	CD69	CD20	CD3
V450	CD3	CD3	CD3	CD3	CD3	CD14
V500	HLA-DR	HLA-DR	HLA-DR	CD4	IgD	HLA-DR

Tfh; follicular helper T cells, DC; Dendritic cell.

Supplementary Table S11. The antibodies used in this study

	clone	Company name	Location
APC-H7 Mouse Anti-Human CD3	SK7	BD Pharmingen	Franklin Lakes, NJ
V450 Mouse Anti-Human CD3	UCHT1	BD Pharmingen	Franklin Lakes, NJ
V500 Mouse Anti-Human CD4	RPA-T4	BD Horizon	Franklin Lakes, NJ
PerCP-Cy5.5 Mouse Anti-Human CD4	RPA-T4	BD Pharmingen	Franklin Lakes, NJ
APC-H7 Mouse Anti-Human CD8	SK1	BD Pharmingen	Franklin Lakes, NJ
PE-Cy7 Mouse Anti-Human CD11c	B-ly6	BD Pharmingen	Franklin Lakes, NJ
V450 Mouse Anti-Human CD14	MφP9	BD Pharmingen	Franklin Lakes, NJ
APC Mouse Anti-Human CD16	B73.1	BD Pharmingen	Franklin Lakes, NJ
FITC Mouse anti-Human CD19	HIB19	BD Pharmingen	Franklin Lakes, NJ
APC-H7 Mouse Anti-Human CD19	SJ25C1	BD Pharmingen	Franklin Lakes, NJ
PerCP-Cy5.5 Mouse Anti-Human CD19	SJ25C1	BD Bioscience	Franklin Lakes, NJ
APC-H7 Mouse Anti-Human CD20	2H7	BD Pharmingen	Franklin Lakes, NJ
PE Mouse Anti-Human CD24	ML5	BD Pharmingen	Franklin Lakes, NJ
PE Mouse Anti-Human CD25	M-A251	BD Pharmingen	Franklin Lakes, NJ
PE-Cy7 Mouse Anti-Human CD27	O323	eBioscience	Waltham, MA
APC Mouse Anti-Human CD38	HIT2	BD Pharmingen	Franklin Lakes, NJ
PE-Cy7 Mouse Anti-Human CD45RA	HI100	BD Pharmingen	Franklin Lakes, NJ
APC-H7 Mouse Anti-Human CD45RO	UCHL1	BD Pharmingen	Franklin Lakes, NJ
PE Mouse Anti-Human CD56	B159	BD Pharmingen	Franklin Lakes, NJ
APC-H7 Mouse Anti-Human CD69	FN50	BD Pharmingen	Franklin Lakes, NJ
PerCP-Cy™5.5 Mouse Anti-Human CD123	7G3	BD Biosciences	Franklin Lakes, NJ
APC Mouse CD127 APC	eBioRDR5	eBioscience	Waltham, MA
PE-Cy7 Mouse Anti-Human CCR4	1G1	BD Pharmingen	Franklin Lakes, NJ
PE-Cy7 Mouse Anti-Human CCR6	11A9	BD Pharmingen	Franklin Lakes, NJ
PE Mouse anti-Human CCR7	150503	BD Pharmingen	Franklin Lakes, NJ
PerCP-Cy5.5 Mouse Anti-Human CXCR3	1C6/CXCR3	BD Pharmingen	Franklin Lakes, NJ
PE Mouse anti-Human CXCR5	#51505	R&D Systems, Inc.	Minneapolis, MN
V500 Mouse anti-Human HLA-DR	G46-6	BD Horizon	Franklin Lakes, NJ
APC Mouse Anti-human ICOS	ISA-3	eBioscience	Waltham, MA
V500 Mouse Anti-Human IgD	IA6-2	BD Horizon	Franklin Lakes, NJ

Supplementary Figure S1. Heat maps produced by cluster analysis of immune cells at baseline

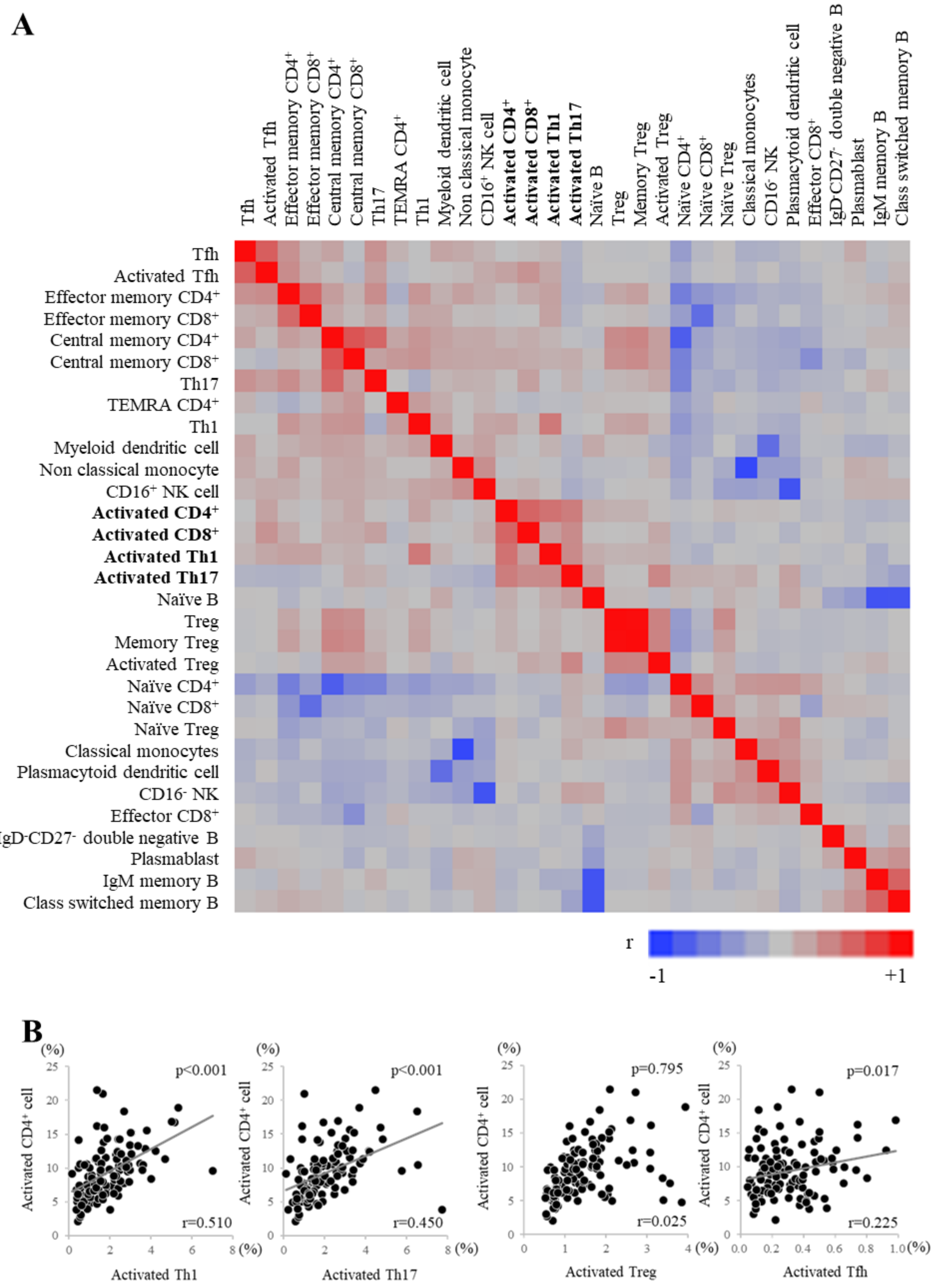

Supplementary Fig. 1.

Pearson product-moment correlation coefficients for the frequencies of immune cells were calculated for the differentiation stage classified as shown as correlation clustering. The heat map of the correlation coefficients summarizes the pairwise correlative relationships (blue for negative correlation and red for positive correlation)

Supplementary Figure S2. Identification of immune cell subsets by eight-colour antibody staining

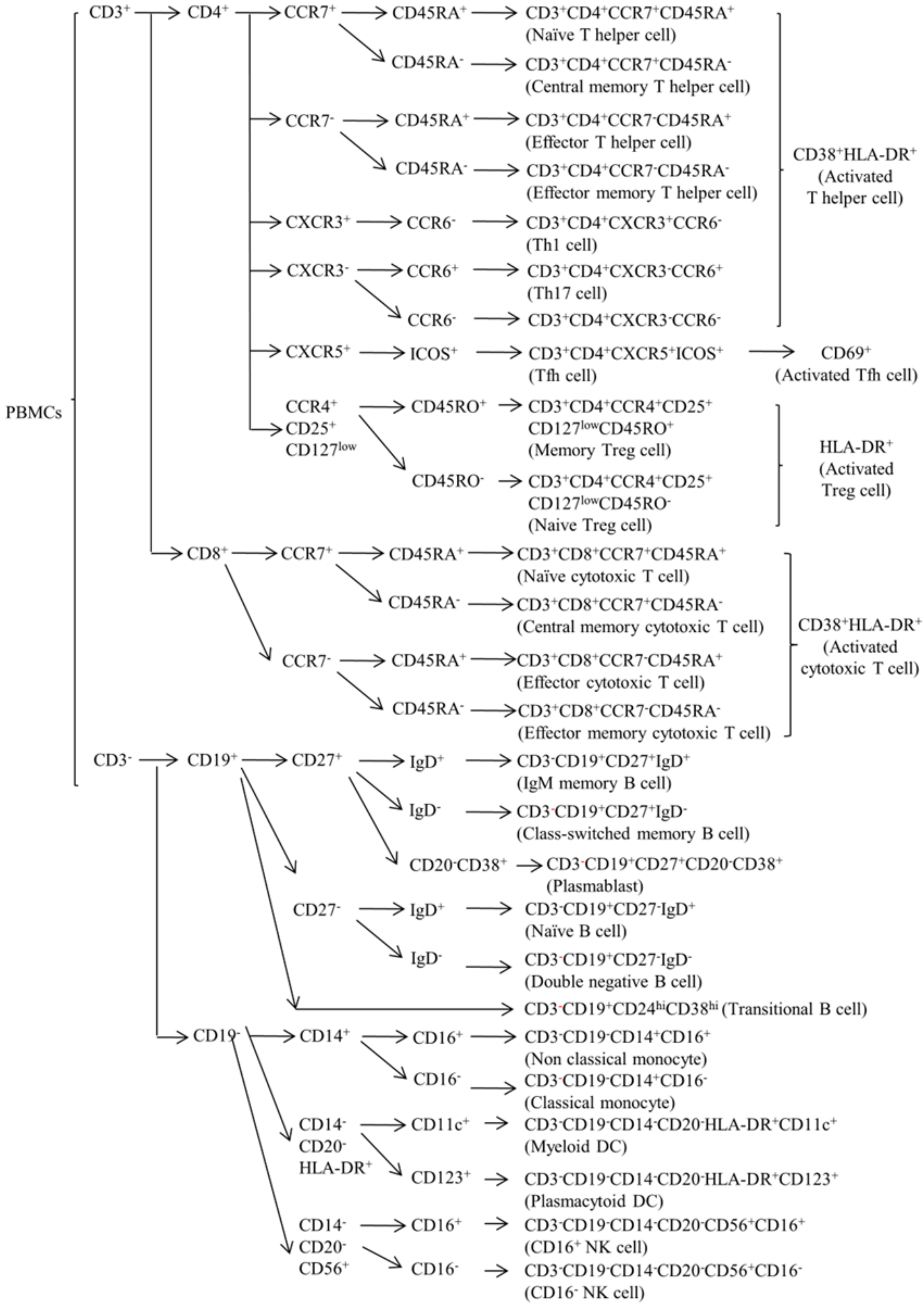

Supplementary Figure S3. Identification of immune cell subsets by eight-colour antibody staining

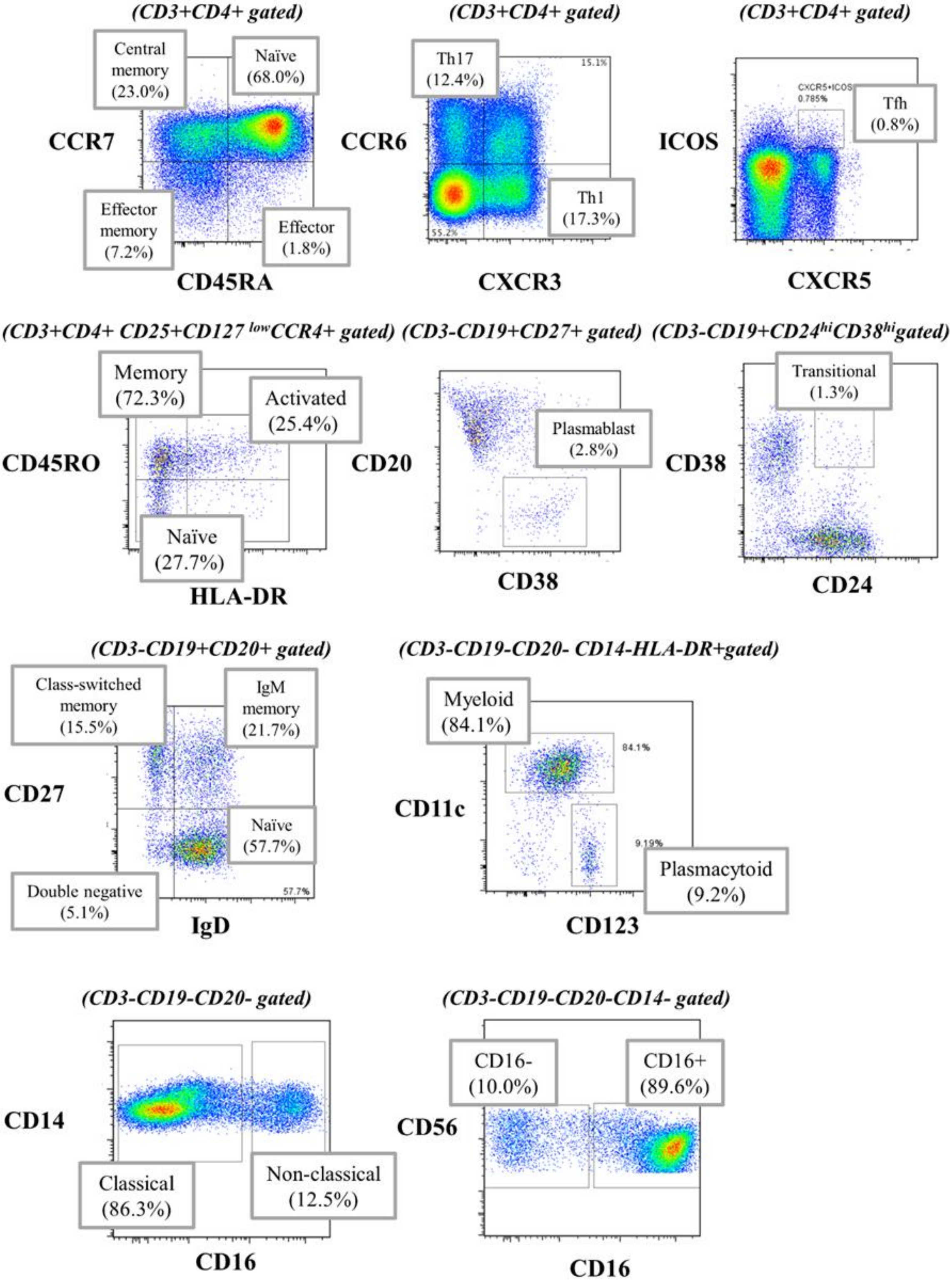

Supplement: Supplementary file 1 — Supplementary Information. [file 41598_2022_19556_MOESM1_ESM.pdf]
